# Identification of Candidate Genes and Regulatory Factors Underlying Intramuscular Fat Content Through Longissimus Dorsi Transcriptome Analyses in Heavy Iberian Pigs

**DOI:** 10.3389/fgene.2018.00608

**Published:** 2018-12-04

**Authors:** María Muñoz, Juan María García-Casco, Carmen Caraballo, Miguel Ángel Fernández-Barroso, Fernando Sánchez-Esquiliche, Fernando Gómez, María del Carmen Rodríguez, Luis Silió

**Affiliations:** ^1^Centro I+D en Cerdo Ibérico INIA-Zafra, Zafra, Spain; ^2^Departamento de Mejora Genética Animal, Instituto Nacional de Investigación y Tecnología Agraria y Alimentaria, Madrid, Spain; ^3^Sánchez Romero Carvajal, Jabugo, Spain

**Keywords:** transcriptome, longissimus dorsi muscle, intramuscular fat, Iberian pigs, RNA-seq

## Abstract

One of the most important determinants of meat quality is the intramuscular fat (IMF) content. The development of high-throughput techniques as RNA-seq allows identifying gene pathways and networks with a differential expression (DE) between groups of animals divergent for a particular trait. The Iberian pig is characterized by having an excellent meat quality and a high content of intramuscular fat. The objectives of the present study were to analyze the longissimus dorsi transcriptome of purebred Iberian pigs divergent for their IMF breeding value to identify differential expressed genes and regulatory factors affecting gene expression. RNA-seq allowed identifying ∼10,000 of the 25,878 annotated genes in the analyzed samples. In addition to this, 42.46% of the identified transcripts corresponded to newly predicted isoforms. Differential expression analyses revealed a total of 221 DE annotated genes and 116 DE new isoforms. Functional analyses identified an enrichment of overexpressed genes involved in lipid metabolism (*FASN, SCD, ELOVL6, DGAT2, PLIN1, CIDEC*, and *ADIPOQ*) in animals with a higher content of IMF and an enrichment of overexpressed genes related with myogenesis and adipogenesis (*EGR1, EGR2, EGR3, JUNB, FOSB*, and *SEMA4D*) in the animals with a lower content of IMF. In addition to this, potential regulatory elements of these DE genes were identified. Co-expression networks analyses revealed six long non-coding RNAs (lncRNAs) (*ALDBSSCG0000002079, ALDBSSCG0000002093, ALDBSSCG0000003455, ALDBSSCG0000004244, ALDBSSCG0000005525*, and *ALDBSSCG0000006849)* co-expressed with *SEMA4D* and *FOSB* genes and one (*ALDBSSCG0000004790*) with *SCD, ELOVL6, DGAT2, PLIN1*, and *CIDEC*. Analyses of the regulatory impact factors (RIFs) revealed 301 transcriptionally regulatory factors involved in expression differences, with five of them involved in adipogenesis (ARID5B, CREB1, VDR, ATF6, and SP1) and other three taking part of myogenesis and development of skeletal muscle (ATF3, KLF11, and MYF6). The results obtained provide relevant insights about the genetic mechanisms underlying IMF content in purebred Iberian pigs and a set of candidate genes and regulatory factors for further identification of polymorphisms susceptible of being incorporated in a selection program.

## Introduction

Intramuscular fat content (IMF) plays a major role in the determination of meat quality since it influences technological and sensorial features such as flavor, tenderness, and juiciness (Verbeke et al., 1999; Wood et al., 2008). Therefore, understanding of the genetic and physiological mechanisms affecting IMF, as well as different gene expression patterns along development has become one of the main challenges in meat science (Hocquette et al., 2010). IMF is strongly influenced by diet, breed and age and it changes across the tissues. The number (hyperplasia) and size (hypertrophia) of adipocytes within the muscle are some of the main determinants of IMF (Shi-Zheng and Su-Mei, 2009). The differentiation of preadipocytes into adipocytes starts during the embryonic growth and continues immediately after birth, to then slow down during the growth of the animal (Sepe et al., 2010). Nonetheless, the increase in adipose cell number and size continues along after the birth and is maintained during all the development of the individual (Lee and Kauffman, 1974). The hypertrophy process is caused by the accumulation of triglycerides in mature adipocytes, which is determined by the ratio between lipogenesis and lipolysis.

Transcriptome studies comparing individuals with extreme phenotypes of a trait are very useful to identify gene pathways and networks with divergent expression among groups for livestock species as cattle or pigs. Particularly, for IMF, these studies contributed to our knowledge of the processes implicated in its deposition (Cánovas et al., 2010; Damon et al., 2012; Hamill et al., 2013; Cesar et al., 2015). In the last years, most of the transcriptome studies have been carried out using high-throughput RNA sequencing (RNA-seq). Unlike expression microarrays, RNA-seq allows the identification and characterization of the full set of transcripts expressed in a tissue as well as the quantification of these transcripts.

The Iberian breed is the most representative of the Mediterranean traditional pig breeds. It is characterized for having lower growth rate, higher adipogenic potential and higher meat quality with respect of other porcine breeds. Its traditional outdoor production system includes a free-range finish-fattening period based on *ad libitum* intake of acorn and grass which strengthens the accumulation of subcutaneous and intramuscular fat (Lopez-Bote, 1998).

Intramuscular fat content is a complex trait with heritability values estimates ranging from 0.26 to 0.69 in Iberian breed populations (García-Casco et al., 2014; Ibáñez-Escriche et al., 2016; Muñoz et al., 2018). This highly hereditary component makes IMF suitable to be included in selection programs focused on obtaining highly prized pig meat and derived products. Since 2012, a breeding program of Iberian pigs fattened under a free-range feeding system is being developed. The main goals of this breeding program are the improvement of premium-cuts yield and meat quality (Muñoz et al., 2018).

Previous transcriptome studies using RNA-seq have revealed very relevant results about genetic expression patterns and networks underlying IMF at different ages, breeds, and tissues (Ayuso et al., 2015, 2016; Cardoso et al., 2018; Li et al., 2018). However, due to the particular characteristics of the Iberian pig to analyze changes in the transcriptome and regulatory factors of finishing Iberian pigs divergent for IMF content is particularly interesting.

In the present study, longissimus dorsi (LD) transcriptomes of two groups of Iberian purebred pigs divergent for IMF breeding values were analyzed with the following objectives. (i) Characterize the LD transcripts of finishing Iberian pigs; (ii) evaluate the expression differences among divergent groups and identify networks and pathways in which the differentially expressed genes are involved; (iii) identify regulatory factors affecting gene expression; (iv) detect potential candidate genes affecting IMF in order to be included to the aforementioned selection scheme.

## Materials and Methods

### Animal Material

Animal manipulations were carried out according to the regulations of the Spanish Policy for Animal Protection RD1201/05, which meets the European Union Directive 86/609 about the protection of animals used in research. Research protocols were assessed and approved by the “Instituto Nacional de Investigación y Tecnología Agraria y Alimentaria” (INIA) Committee of Ethics in Animal Research, which is the named Institutional Animal Care and Use Committee (IACUC) for the INIA. The animal material used in the present study belongs to a closed commercial population of Iberian purebred pigs in which the individuals were fed under a restricted feeding regime up to a live weight of approximately 100 kg and fattened in a free-range system based on the *ad libitum* intake of acorns and grass. Castrated males were slaughtered at an approximate age of 17 months and a final weight of 165 kg. IMF was measured as a percentage using infrared spectroscopy (NIRS) in LD samples of 914 animals slaughtered in 15 batches. The mean of the IMF was 5.11 (*SD* = 2.08).

The following mixed model was used in order to estimate breeding values (EBVs) for IMF:

$$y=\mathrm{Xb}+\mathrm{Za}+W_{\mathrm{sm}}\mathrm{sm}+W_{1}1+\mathrm{e,}$$

where **y** is the IMF of each animal, **b** represents the systematic effects in which the slaughter weight was fitted as a covariate, **a** is the vector of the additive genetic effects, **sm** is the vector of the combined free-range*/*slaughter batches random effects (28 levels), **l**, the vector of the litter random effect and **e**, the vector including the residual effects. **X**, **Z**, **W_**sm**_**, and **W_**l**_** are the incidence matrices. EBVs were estimated using the TM program (Legarra et al., 2008).

These data were used to select the pigs with the most extreme EBVs for IMF. The selection included castrated males belonging to 10 combined free-range*/*slaughter batches and excluding full and half siblings. The 12 animals with extreme EBVs were selected. The six individuals with highest values were included in the high group (H) and the six individuals with lowest values; in the low group (L). The mean percentages of IMF were 11.95 (*SD* = 1.25) and 3.45 (*SD* = 0.26) for the H and the L group, respectively, and the corresponding averaged EBVs were 3.07 (*SD* = 0.32) and -1.38 (*SD* = 0.05).

### Transcriptomic Analyses

#### RNA Extraction

Samples of LD of the selected 12 pigs were collected at slaughter, frozen in liquid nitrogen and stored at -80°C until analyzed. Total RNA was extracted using RiboPure^TM^ of High Quality total RNA kit (Ambion, Austin, TX, United States) following the manufacturer’s recommendations. The RNA integrity was assessed using the RNA Integrity Number (RIN) value from the Agilent 2100 Bioanalyzer device (Agilent technologies, Santa Clara, CA, United States). RNA integrity values ranged between 7 and 8.

#### Library Preparation and Sequencing

Paired-end libraries were prepared using TruSeq SBS Kit v3-(Illumina, San Diego, CA, United States) for each sample. Multiplex sequencing of the libraries was carried out on a HiSeq 2000 sequence analyzer (Illumina, Inc.) with four samples per lane, according to the manufacturer’s instruction at Centro Nacional de Análisis Genómico. Paired-end reads of 76 bp were generated. The raw sequence data have been deposited in the Gene Expression Omnibus (GEO) expression database under the accession number: GSE116951.

#### Mapping and Assembly

FastQC was used to evaluate the quality of the raw sequencing data^1^. The quality was measured according to sequence-read lengths and base-coverage, nucleotide contributions and base ambiguities, quality scores and over-represented sequences. All the samples passed the QC parameters; they had the same length, 100% coverage in all bases, 25% of A, T, G, and C nucleotide contributions, 50% GC on base content and less than 0.1% of over-represented sequences. The sequences were trimmed to remove the sequencing adaptor and poly A and T tails with Trim Galore^2^ setting default values (stringency of 6 bp) and paired-end reads where kept when both pairs were longer than 40 bp. Filtered reads were mapped against the pig reference genome (Sscrofa11.1) using TopHat v2.1.0 (Trapnell et al., 2009) applying default settings, except the reads were aligned first to the ENSEMBLE (Sscrofa11.1) transcriptome annotation (-G option) and the distance between pairs was set to 50 bp (inner-mean distance) and the standard deviation at 150 bp. Transcripts were assembled and quantified in Fragments Per Kilobase Million (FPKM) using Cufflinks v2.2.1 (Trapnell et al., 2012). The normalized expression data have been deposited in the GEO database with the accession number GSE116951. In addition, the CummeRbund Bioconductor R package (Goff et al., 2013) was used to determine the clustering of the samples according to their expression data and assess the consistence of the group analyzed.

#### Identification of Novel Isoforms and Long Non-coding RNAs (lncRNAs)

Isoforms not described so far were extracted using Cuffcompare tool from Cufflinks. Cuffcompare was run using ENSEMBL (Sscrofa11.1) transcriptome annotation as a reference to assess the accuracy of the predicted Cufflinks mRNAs or gene models and reducing the set of reference transcripts to only those overlapping with any of the input loci.

The domestic-animal lncRNA database (ALDB) (Li et al., 2015) was used to identified the long non coding RNAs (lncRNAs). Mapping and assembly were re-run using Pig lncRNAs annotation^3^.

#### Differential Expression Analyses

The expression values and the differential expression analyses between the H and L groups of the annotated genes and newly described isoforms were carried out with the Cuffdiff tool. The bias correction (-b option) and the rescue method for multireads (-u option) were set for running Cuffdiff and the remaining parameters were established as default. Two criteria were used to filter the genes, newly predicted isoforms and lncRNAs: a minimum mean group expression of 0.5 FPKM and a value of the log_2_ fold change (log_2_FC) of the expression differences among H and L equal to 1.2. In addition, to correct multiplicity of test, R package *q*-value (Storey and Tibshirani, 2003) was used, those genes and new isoforms were considered as differentially expressed (DE) with a *p-*value of 0.05 and *q*-value of 0.10.

#### Gene Functional Classification, Network and Pathway Analyses

Functional analyses of the differential expressed genes between H and L groups were carried out with the FatiGO browser from Babelomics 5^4^ using Gene Ontology (GO) database and Genome-Scale Metabolic Network (Recon). STRING tools (Szklarczyk et al., 2017) were used to study the potential interactions between the proteins codified by the DE genes and clustering through Markov Cluster Algorithm (MCL). Additionally, Ingenuity Pathway Analysis (IPA) (Ingenuity Systems, Qiagen, CA, United States) software was used to identify and characterize biological functions, gene networks and canonical pathways affected by the DE genes.

#### Correlation Among DE lncRNAs and Genes in IPA Networks

Correlations between expression values of DE lncRNAs and genes involved in the three top IPA networks were calculated. A confidence interval (CI) was obtained by bootstrapping. The correlation between randomly selected lncRNA and one gene from a total of 4,535 lncRNAs and 8,639 gene transcripts was calculated in each iteration of bootstrapping. A total of 10,000 iterations were carried out to estimate the 95% CI. Significant correlations were represented using the software Cytoscape 3.5.1 (Shannon et al., 2003). Subsequently, the location of the lncRNAs and the significantly correlated genes of the IPA Network 3 (NW3) were analyzed. As the annotation of the lncRNAs was not available for the Sscrofa11.1 annotation version, the location of the closest gene of a determined lncRNA was searched in Sscrofa10.2.

A weighted gene co-expression network analysis was performed on the DE lncRNAs and genes belonging to the IPA NW3 with the R package WGCNA (Langfelder and Horvath, 2008). The weighted correlation network was built through the creation of a matrix of pairwise Pearson correlation coefficients. A value of 6 was chosen as a soft-threshold and made the adjacency network exhibit scale-free topology. The topological overlap matrix was constructed based on the adjacency matrix. After that, the genes were clustered into different modules using hierarchical clustering and dynamic tree cutting. According to the WGCNA terminology, the first principal component was defined for each module as the eigengene. Correlations between IMF content and eigengenes of each module were calculated. The connectivity of each gene based in their intramodular connectivity was also calculated. lncRNAs with top 20% intramodular connectivity were defined as hub lncRNAs.

### Search of Transcription Factors With RIF Metrics

Regulatory transcription factors (TRF) potentially affecting the observed DE genes were also analyzed. Regulatory Impact Factors (RIF1 and RIF2) (Reverter et al., 2010) were estimated for the set of DE genes related with fatness and skeletal muscle development (139). RIF1 assigns an extreme score to the TRF consistently most differentially co-expressed with the highly abundant and highly DE genes, in addition to this, RIF2 assigns an extreme score to TRF with the most altered ability acting as predictor of the abundance of DE genes. A list of 1834 candidate TRFs were obtained from the Transcription factor prediction database^5^. Among them, 1,602 showed expression values greater than 0.5 FPKM in at least one experimental group and they were used in the RIF approach. RIF1 and RIF2 values for the *i*_th_ TRF were calculated as follows:

$$\mathrm{RIF}1_{i}=\frac{1}{n_{\mathrm{DE}}}\sum_{j=1}^{j=n_{\mathrm{DE}}}\hat{a_{j}}x\hat{d_{j}}{\left(r1_{\mathrm{ij}}-r2_{\mathrm{ij}}\right)}^{2}$$

$$\mathrm{RIF}2_{i}=\frac{1}{n_{\mathrm{DE}}}\sum_{j=1}^{j=n_{\mathrm{DE}}}\left\{{\left(e1_{j}{\mathrm{xrl}}_{\mathrm{ij}}\right)}^{2}-{\left(e2_{j}\mathrm{xr}2_{\mathrm{ij}}\right)}^{2}\right\}$$

where n_DE_ is the number of DE genes, a_j_ and d_j_, the estimated average expression and differential expression of the *j*_th_ DE gene, r1_ij_ and r2_ij_ the co-expression correlation between the *i*_th_ TRF and the *j*_th_ DE gene in the H and L groups. In addition, *e*1j and *e*2j represent the expression of the *j*_th_ DE gene in the H and L groups, respectively. Both RIFs measures were transformed to *z*-scores by subtracting the mean and dividing it by the standard deviation. Relevant TRF were identified as those with extreme values according to their corresponding confidence intervals (CI) calculated by bootstrap. In each iteration of bootstrapping, a set of *n*_DE_ = 139 genes were randomly selected from a total of 8,639 transcripts with expression values greater than 0.5 FPKM in at least one experimental group and the RIF1 and RIF2 *z*-scores of the 1602 TRF were calculated. To estimate the 99% CI intervals, this procedure was repeated 10,000 times.

The IPA software was also used to characterize and identify potential TRFs and using Genomatix software suite^6^, presence of Transcription Factor Binding Sites (TFBS) was checked in the promotor sequences of DE genes, which could bind the predicted TRFs. The Mathinspector tool identifies TFBS in sequences using a large library of weight matrices (Matrix Library 11.0). For each matrix family Mathinspector lists how many matches are found in total, in how many sequences and how often it matches in each input sequence, in addition to this, it gives a *p-*value for each common TF site. This *p-*value is the probability to obtain an equal or greater number of sequences with a match in a randomly drawn sample of the same size as the input sequence set.

### Validation by qPCR of Results

RNA samples from the 12 selected animals were used to carry out the technical validation of the differential expression of some genes that were upregulated in the H group, in L group or not DE between groups. First-strand cDNA synthesis was performed using Superscript II (Invitrogen, Life Technologies, Paisley, United Kingdom) and random hexamers in a total volume of 20 μl containing 1 μg of total RNA and following the supplier’s instructions. The expression of 15 genes was quantified by qPCR. Primer pairs used for quantification were designed using Primer Select software (DNASTAR, Madison, WI, United States) and/or Primer-Blast^7^ from the available GENBANK and/or ENSEMBL sequences, covering different exons in order to assure the amplification of the cDNA. Supplementary Table 1 shows primer sequences. First, standard PCRs on cDNA were carried out to verify amplicon sizes. Quantification was done using SYBR Green Mix (Roche, Basel, Switzerland) in a LightCycler^®^ 480 (Roche, Basel, Switzerland), following standard procedures and data were analyzed with LightCycler^®^ 480 SW1.5 software (Roche, Basel, Switzerland). All the samples were run three times and dissociation curves were obtained for each individual replicate. Single peaks in the dissociation curves confirmed the specific amplification of the genes. For each gene, PCR efficiency was estimated using the standard curve calculation using four points of the cDNA serial dilutions. Mean *C*p values were used for the statistical analyses. Stability of *B2M* and *TBP* endogenous genes was calculated using GeNorm software (Vandesompele et al., 2002) and they were used to normalize the data. Normalization factors were used to carry out the data normalization. Relative quantities were divided by the normalization factors, which were the geometric means of the two reference gene quantities. The technical validation was performed by calculating Pearson correlation between the expression values from RNA-seq data (FPKM) and the normalized gene expression data of the qPCR and estimating the concordance correlation coefficient (CCC) (Miron et al., 2006) between the FC values estimated from RNA-seq and qPCR expression measures for the 15 genes analyzed by the two technologies (RNA-seq and qPCR).

## Results and Discussion

### Characterization of Longissimus Dorsi Transcriptome and Differential Expression Analyses

A total of 1,630 million of paired-end reads were obtained from the LD transcriptome sequencing of the 12 samples. After trimming and filtering, 1,623 million reads remained. Between 94.7 and 95.5% of the reads were mapped against the pig reference genome across samples (Supplementary Table 2). These percentages were higher than previously reported values in other studies: 55.5–63.0% (Pérez-Montarelo et al., 2014), 76.5–86.6% (Puig-Oliveras et al., 2014), 67.0–77.0% (Ayuso et al., 2015), all of them were mapped using the previous version of the porcine reference genome (*Sscrofa*10.2). Therefore, these results seem to confirm a greater accuracy of the new version of the reference genome comparing with the previous one.

The Cufflinks tool revealed a total of 137,022 transcripts expressed in the 12 samples. Table 1 summarizes the classification carried out with Cuffcompare. 8.96% of the transcripts were annotated as intergenic. This value was lower than those observed in other studies using an experimental backcross Iberian × Landrace in LD, 10.2% (Puig-Oliveras et al., 2014) and other tissues: 20% in adipose tissue (Corominas et al., 2013) and 27.8% in hypothalamus (Pérez-Montarelo et al., 2014) using the previous ENSEMBL transcriptome annotation (*Sscrofa*10.2). However, there were also a high percentage of transcripts falling entirely within a reference intron (16.69%) that could suggest intron retention events, incorrect annotation of exons, errors or missing prediction of isoforms. In addition, the percentage of potentially new isoforms predicted represents the 42.46% of the transcripts detected. This could be due to an incomplete porcine genome annotation which that still does not gather all the isoforms expressed in LD or to an incorrect assembly of the full-length transcript caused by incomplete coverage of the less expressed transcripts (Roberts et al., 2011).

**Table 1:** Classification of the transcripts identified in the longissimus dorsi samples in relation to the Ensembl annotated pig genes.

	Transcripts, *n*	Transcripts %
Complete match of intron chain	12,803	9.34
Contained in the reference	8,492	6.20
Potentially novel isoform	58,182	42.46
Possible pre-mRNA	3,791	2.77
Transcript falling within a reference intron	22,863	16.69
Generic overlap with a reference transcript	2,594	1.84
Possible polymerase run-on fragment	2,151	1.57
Intergenic transcript	12,276	8.96
Exonic overlap on the opposite strand	1,974	1.44
Multiple classifications	11,966	8.73
Total	137,022	100.00


**FIGURE 1:**
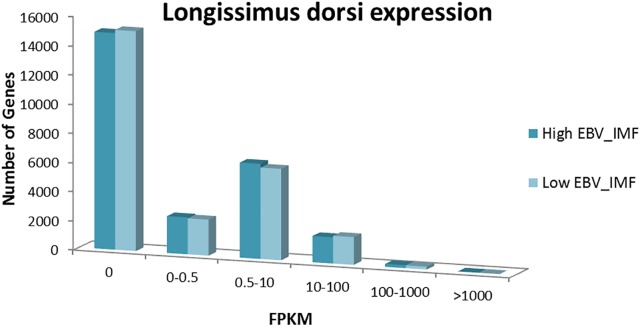
Gene expression distribution of the 25,878 genes annotated in the pig genome (Sscrofa11.1) in fragments per kilobase million (FPKMs) normalized values in the H and L groups.

Figure 1 represents the expression values obtained with Cuffdiff for the 25,878 genes annotated in the pig genome. The distribution of gene expression levels was similar in both groups. A total of 14,837 and 15,053 genes in the H and L groups, respectively, were not expressed, therefore, less than a 50% of total porcine annotated genes in the *Sscrofa*11.1 genome assembly (10,029 on average in the two groups) were expressed in the studied samples. These values were lower than those reported in studies of biceps femori of Iberian purebred and a Duroc × Iberian crossbred piglets, with an average of 11,392 genes expressed out of the 22,861 annotated genes in the Sscrofa10.1 (Ayuso et al., 2015).

The differential expression analyses showed a total of 221 DE of the annotated genes and 116 DE newly predicted isoforms. While 78 of the annotated genes were upregulated in the H group, the remaining 143 genes showed greater expression in the L group. In addition to this, 44 newly predicted isoforms showed higher expression in the H group and 72 in the L group. The log_2_ fold change of these genes ranged from 1.2 to 3.41.

Recent developments in cDNA cloning and sequencing technologies have disclosed a huge number of non-coding transcripts (ncRNAs) of variable length, localization, and function. The current approach for studying ncRNAs aims to further classify these transcripts by size, where ncRNAs longer than 200 nucleotides are referred to as lncRNAs. Through their interactions with proteins, RNA and DNA, lncRNAs could provide various regulatory mechanisms necessary for mammalian development. Frequently, lncRNAs regulates the expression of close genes (Guil and Esteller, 2012) and a number of lncRNAs involved in lipid metabolism and IMF development has been recently identified and characterized in the pig LD transcriptome (Zou et al., 2018). In the present study, a total of 4,506 lncRNAs out of 4,535 were expressed in LD muscle. The differential expression of these lncRNAs showed a total of 43 DE lncRNAs between H and L groups.

### *q-PCR* Validation of Differential Expression Analyses

In order to validate the RNA-seq experiment, real-time quantitative PCR (q-PCR) was used to assess the expression of 15 genes (five upregulated and five downregulated in the H group and five unchanged, two of them showing low expression and the remaining three showing a medium-high expression). All the correlation coefficients between both measures of gene expression were positive and 13 were significantly different from zero (Table 2). The CCC calculated between the expression measures (FC) obtained from RNA-seq and q-PCR (CCC = 0.98) points out a substantial strength-of-agreement of the experiment which validate the global reproducibility of the RNA-seq results. The *SPP1* gene showed the highest concordance between methods and *CNOT10* the one with the lowest agreement.

**Table 2:** Technical validation of RNA-seq experiment by qPCR: list of validated genes, log_2_ of fold change values (log_2_FC) between high (H) and low (L) groups and Pearson correlations between expression values obtained from each technique.

Gene	log_2_FC RNA-seq	log_2_FC qPCR	Pearson correlation	*p-*Value
*ADIPOQ*	1.31	1.33	0.571	0.053
*ATF3*	-1.17	-1.22	0.984	6.92 × 10^-9^
*CNOT10^∗^*	-0.25	-0.14	0.642	0.024
*DCAF5^∗^*	-0.26	0.04	0.821	0.001
*DNAJA1^∗^*	-0.57	-0.53	0.961	1.01 × 10^-8^
*EGR1*	-1.64	-1.71	0.962	5.63 × 10^-7^
*ELOVL6*	1.50	0.83	0.807	0.002
*FASN*	2.54	1.19	0.808	0.001
*FOS*	-1.67	-1.65	0.961	6.72 × 10^-7^
*GPHN^∗^*	-0.05	-0.10	0.550	0.064
*ITGB6^∗^*	0.12	-0.18	0.758	0.004
*PFKFB3*	-1.10	-0.67	0.981	1.83 × 10^-8^
*PLIN1*	1.39	1.51	0.959	8.89 × 10^-7^
*SCD*	1.55	1.58	0.857	3.67 × 10^-7^
*SPP1*	-1.62	-1.57	1.000	6.67 × 10^-18^


*^∗^No differentially expressed in RNA-seq experiment; concordance correlation coefficient (CCC) value = 0.98.*

### Functional Analyses

The GO enrichment analyses carried out with FatiGO (Babelomics) on the 221 DE annotated genes and 116 DE predicted isoforms identified 197 GO biological processes (GO_BP_) and five GO_SLIM_ enriched in DE genes (Supplementary Table 3). Three out of the ten top overrepresented pathways with adjusted *p*-values <10^-7^ involved skeletal muscle tissue: skeletal muscle tissue development (eight genes), skeletal muscle organ development (eight genes), and skeletal muscle cell differentiation (six genes). Among the 197 GO terms, 11 GO_BP_ and one GO_SLIM_ were related with fat metabolism (Table 3). The Genome-Scale Metabolic Network (Recon) revealed an enrichment of genes upregulated in the H group in the *fatty acid elongation* pathway (Table 3) pointing out an overstimulation of the lipogenesis in muscle of animals with high EBVs for IMF.

**Table 3:** Summary of significantly overrepresented pathways related with lipid metabolism in the DE genes between H and L groups.

Term	Genes	Adjusted *p-*value
GO_BP_		
Response to fatty acid (GO:0070542)	*CTGF, ADCY6, ADIPOQ, DGAT2*	1.38 × 10^-4^
Triglyceride metabolic process (GO:0006641)	*ELOVL6, THRSP, FASN, PLIN1, DGAT2*	3.75 × 10^-4^
Neutral lipid metabolic process (GO:0006638)	*ELOVL6, THRSP, FASN, PLIN1, DGAT2*	4.27 × 10^-4^
Fat pad development (GO:0060613)	*ARID5B, DGAT2*	8.77 × 10^-4^
Triglyceride biosynthetic process (GO:0019432)	*ELOVL6, THRSP, FASN, DGAT2*	0.001
Neutral lipid biosynthetic process (GO:0046460)	*ELOVL6, THRSP, FASN, DGAT2*	0.002
Long-chain fatty-acyl-CoA metabolic process (GO:0035336)	*ELOVL6, FASN, DGAT2*	0.002
Fatty-acyl-CoA metabolic process (GO:0035337)	*ELOVL6, FASN, DGAT2*	0.003
Adipose tissue development (GO:0060612)	*ARID5B, DGAT2*	0.003
Long-chain fatty-acyl-CoA biosynthetic process (GO:0035338)	*ELOVL6, FASN*	0.003
Fatty-acyl-CoA biosynthetic process (GO:0046949)	*ELOVL6, FASN*	0.003
GO_SLIM_		
Lipid particle (GO:0005811)	*CIDEC, PLIN1, DGAT2*	0.003
*Recon*		
Fatty_acid_elongation	*FASN, SCD*	0.003


*GO_*BP*_, biological process; GO_*SLIM*_, cut-down versions of the GO ontologies containing a subset of the terms in GO; *Recon*, Genome-Scale Metabolic Network.*

**FIGURE 2:**
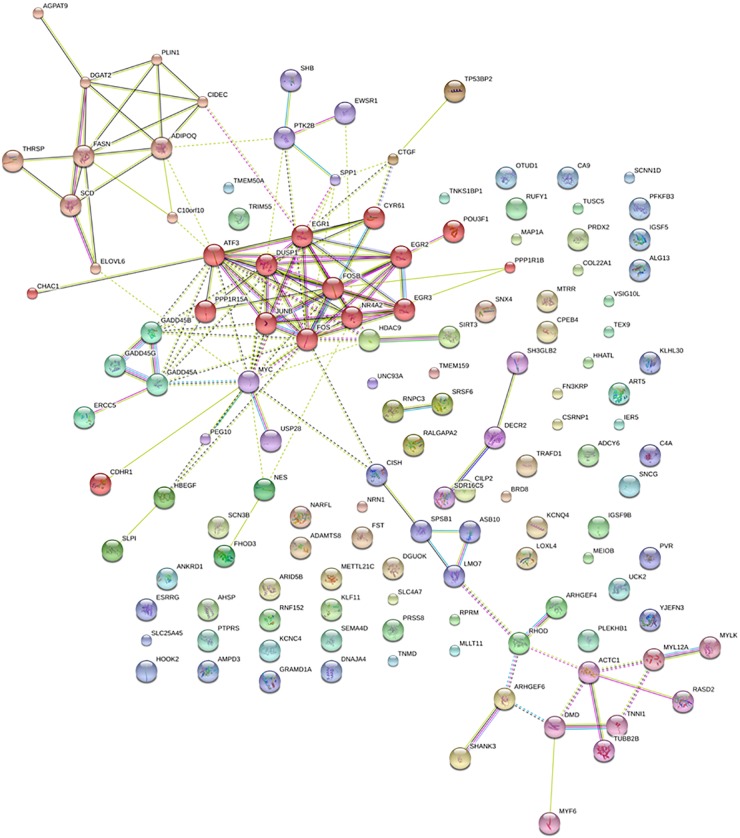
Network of protein–protein interactions predicted with STRING database. Same color nodes sharing multiple edges are grouped in the same cluster.

Markov Cluster Algorithm clustering of the proteins codified by DE annotated and newly predicted isoforms carried out with STRING v10 revealed three main clusters (Figure 2). Cluster 1 comprised AGPAT9, DGAT2, PLIN1, CIDEC, ADIPOQ, FASN, THRSP, SCD, ELOVL6, and *C10orf10*, all related with lipid metabolism; cluster 2, comprised CHAC1, ATF3, PPP1R15A, DUSP1, JUNB, FOS, EGR1, FOSB, NR4A2, CYRS1, EGR2, EGR3, POU3F1, and PPP1R1B related with skeletal muscle development and differentiation and cluster 3 comprised ACTC1, DMD, MYF6, MYL12A, MYLK, RASD2, TNN1, and TUBB2B, some of them are involved in processes as muscle contraction, acto-myosin structure organization or actin filament-based movement.

**FIGURE 3:**
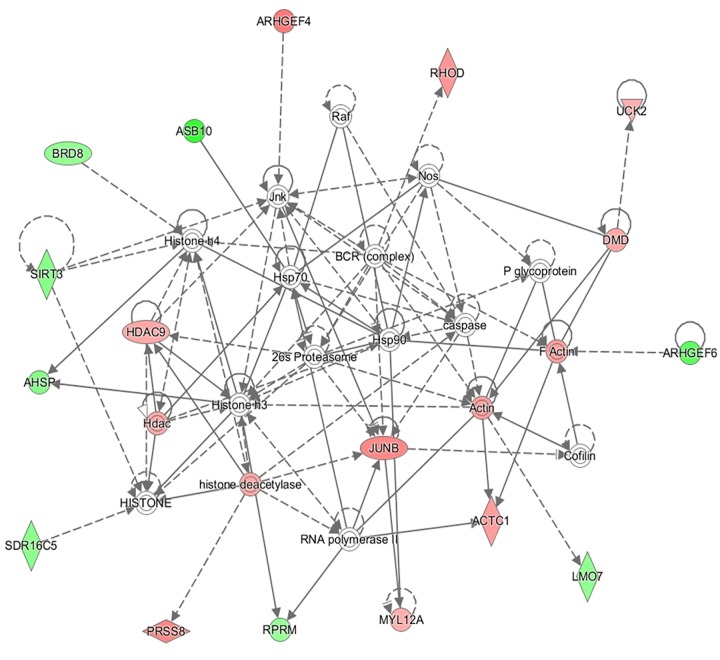
Gene network 1: Cell Morphology, Organ Morphology, Skeletal and Muscular System Development and Function. Genes upregulated and downregulated in the H group are represented by green and red colors, respectively.

**FIGURE 4:**
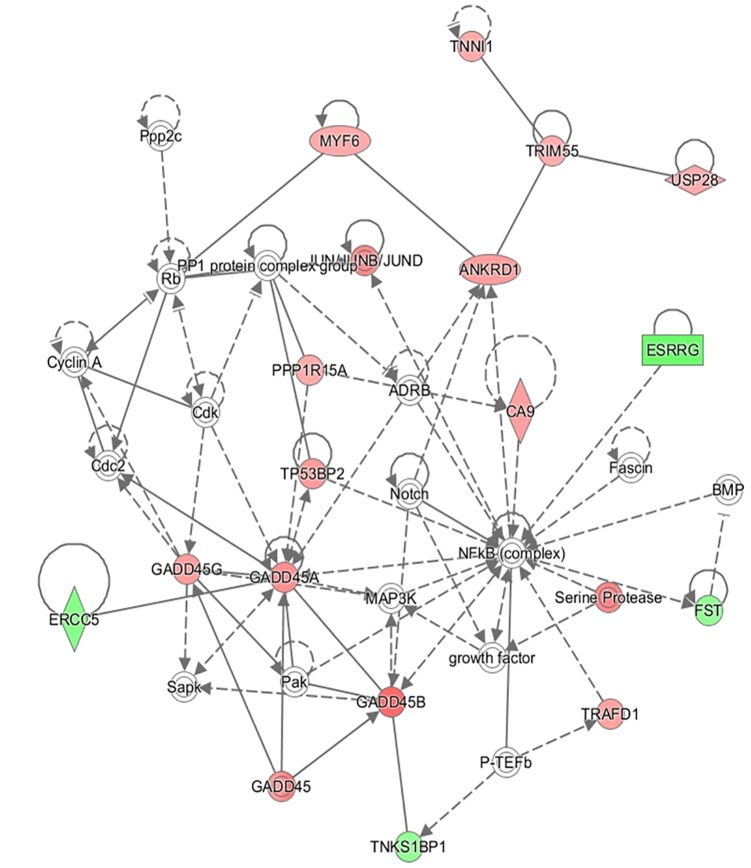
Gene network 2: Cell Cycle, Cellular Growth and Proliferation, Cellular Development. Genes upregulated and downregulated in the H group are represented by green and red colors, respectively.

**FIGURE 5:**
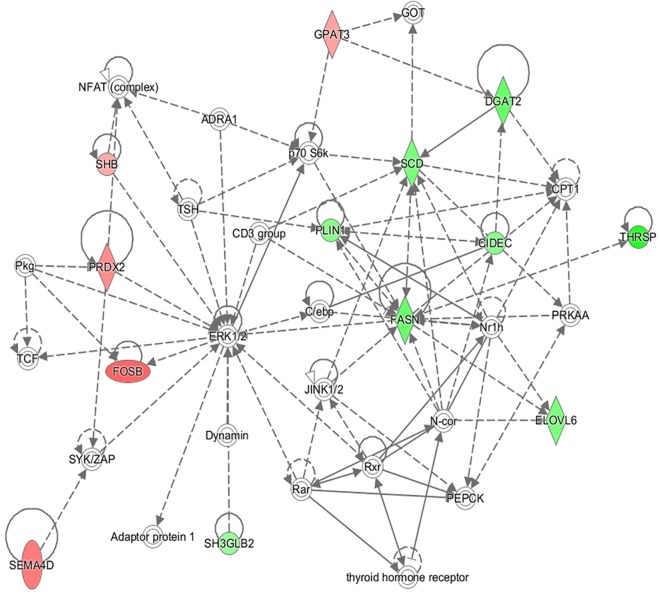
Gene network 3: Lipid Metabolism, Small Molecule Biochemistry, and Carbohydrate Metabolism. Genes upregulated and downregulated in the H group are represented by green and red colors, respectively.

In addition to this, the IPA software was used to build networks with the DE genes. A total of 18 networks were identified. The top three networks are represented in Table 4. Some of the functions represented in gene network 1 (NW1, Figure 3) are related with *Skeletal and Muscular System Development and Function*, in gene network 2 (NW2, Figure 4), with *Cell Cycle and Cellular* Growth, Proliferation and Development while one of the main function represented in gene network 3 (NW3, Figure 5) is *Lipid metabolism*.

**Table 4:** Top three networks enriched with DE genes detected by IPA software.

			Focus	
ID	Molecules in network	Score	molecules	Top functions
1	26S proteasome, **ACTC1**, actin, **AHSP**, **ARHGEF4**, **ARHGEF6**, **ASB10**, BCR (complex), **BRD8**, caspase, cofilin, **DMD**, F actin, Hdac, **HDAC9**, HISTONE, histone deacetylase, Histone h3, Histone h4, Hsp70, Hsp90, Jnk, **JUNB**, **LMO7**, **MYL12A**, Nos, P glycoprotein, **PRSS8**, Raf, **RHOD**, RNA polymerase II, **RPRM**, **SDR16C5**, **SIRT3**, **UCK2**	31	17	Cell Morphology, Organ Morphology, Skeletal and Muscular System Development and Function
2	ADRB, **ANKRD1**, BMP, **CA9**, Cdc2, Cdk, Cyclin A, **ERCC5**, **ESRRG**, Fascin, **FST**, GADD45, **GADD45A**, **GADD45B**, **GADD45G**, growth factor, JUN/JUNB/JUND, MAP3K, **MYF6**, NF-κB (complex), Notch, P-TEFb, Pak, PP1 protein complex group, **PPP1R15A**, Ppp2c, Rb, Sapk, Serine Protease, **TNKS1BP1**, **TNNI1**, **TP53BP2**, **TRAFD1**, **TRIM55**, **USP28**	29	16	Cell Cycle, Cellular Growth and Proliferation, Cellular Development
3	Adaptor protein 1, ADRA1, C/ebp, CD3 group, **CIDEC**, CPT1, **DGAT2**, dynamin, **ELOVL6**, ERK1/2, **FASN**, **FOSB**, GOT, **GPAT3**, JINK1/2, N-cor, NFAT (complex), Nr1h, p70 S6k, PEPCK, Pkg, **PLIN1**, **PRDX2**, PRKAA, Rar, Rxr, **SCD**, **SEMA4D**, **SH3GLB2**, **SHB**, SYK/ZAP, TCF, **THRSP**, thyroid hormone receptor, TSH	22	13	Lipid Metabolism, Small Molecule Biochemistry, and Carbohydrate Metabolism


*DE genes between H and L groups in **bold**.*

The most relevant genes showing a higher expression in the H group correspond to biological processes related with lipogenesis and lipid storage (Figure 5). *Fatty acid synthase* (*FASN*) gene (log_2_FC = 1.73, *p-*value = 5 × 10^-5^) codifies for the key enzyme in the *de novo* synthesis of fatty acids from palmitic fatty acid (C16:0) (Tehlivets et al., 2007). Then, the product of the*, fatty acid elongase 6* (*ELOVL6*) gene (log_2_FC = 1.50, *p-*value = 0.001) elongates 16 and 18 carbon molecules (Moon et al., 2001) and the protein coded by the *stearoyl-CoA desaturase* (*SCD*) gene (log_2_FC = 1.55, *p*-value = 5 × 10^-5^) plays a key role in the desaturation of fatty acids and synthesis of oleic fatty acid (C18:1). An overexpression of these genes in muscle of animals with a higher content in IMF has been shown in other studies (Cánovas et al., 2010; Hamill et al., 2013; Puig-Oliveras et al., 2014; Cardoso et al., 2018) and an overexpression of *SCD* gene in muscle has been related with a high lipid storage (Flowers and Ntambi, 2008). *Diacylglycerol O-acyltransferase 2* (*DGAT2*) gene is also upregulated in the H group (log_2_FC = 1.76, *p-*value = 5 × 10^-5^). The protein codified by this gene also promotes *de novo* lipogenesis since is involved in triglycerides synthesis and favors lipid storage. In addition to this, *perilipin 1* (*PLIN1*) gene (log_2_FC = 1.51, *p-*value = 0.009) codifies for a protein belonging to the family of perilipins which play a role in regulating intracellular lipid storage and mobilization (Bickel et al., 2009). Other authors reported a higher expression of *perilipin 2* (*PLIN2*) in muscle with a higher IMF content, however, no differences in expression were reported for *PLIN1* (Gandolfi et al., 2011). *Cell death inducing DFFA like effector c* (*CIDEC*) gene (log_2_FC = 1.40, *p-*value = 0.010) encodes for a protein implied in lipid droplet formation in adipocytes and it also has higher expression in muscle of animals with high IMF content both in pig (Puig-Oliveras et al., 2014) and cattle (Hudson et al., 2015). Besides the genes of the NW3, *Adiponectine* (*ADIPOQ*) gene codifies for a protein (log_2_FC = 1.31, *p-*value = 5 × 10^-5^) involved in the control of fat metabolism and insulin sensitivity and overexpression of this gene in *gluteus medius* was also observed in Duroc pigs with a high content in intramuscular fat (Cánovas et al., 2010).

Generally, pigs with a high muscularity display a lower content in IMF (Hocquette et al., 2010). This agrees with the negative genetic correlation (-0.29 ± 0.11) between IMF and loin percentage on carcass observed in this Iberian pig population. In the present study genes involved in muscle growth and development (GO:0007519, GO:0060538, GO:0035914, Supplementary Table 3) are under-regulated in the H group. *Early growth factors* genes *EGR1* (log_2_FC = -1.63, *p-*value = 5.00 × 10^-5^), *EGR2* (log_2_FC = -1.67, *p-*value = 5.00 × 10^-5^) and *EGR3* (log_2_FC = -1.39, *p-*value = 5.00 × 10^-5^) codify for transcription factors of the EGR family. Previous studies have demonstrated that both *EGR1* and *EGR2* are involved in the regulation of adipogenesis (Boyle et al., 2009), however, their exact role in IMF deposition requires further investigations (Wang et al., 2017). Another group of DE genes encode for TF involved in myogenesis, *Jun Proto-* *JunB* *proto-Oncogene, AP-1 Transcription Factor Subunit* (*JUNB*) (log_2_FC = -1.79, *p-*value = 5.00 × 10^-5^), *Fos Proto-Oncogene, AP-1 Transcription Factor Subunit* (*FOS*) (log_2_FC = -1.67, *p-*value = 5.00 × 10^-5^) and *FosB Proto-Oncogene, AP-1 Transcription Factor Subunit* (*FOSB*) (log_2_FC = -2.30, *p-*value = 5.00 × 10^-5^) genes encoded for transcription factors involved in myogenesis (Trouche et al., 1995; Ponsuksili et al., 2015). *Semaphorin-4d* (*SEMA4D*) gene was also under-regulated in the H group (log_2_FC = -1.96, *p-*value = 0.005), other authors have shown that the knock-down *SEMA4D* zebrafish developed abnormal trunks due to the prolonged expression of myogenic factors (Yang et al., 2013).

**FIGURE 6:**
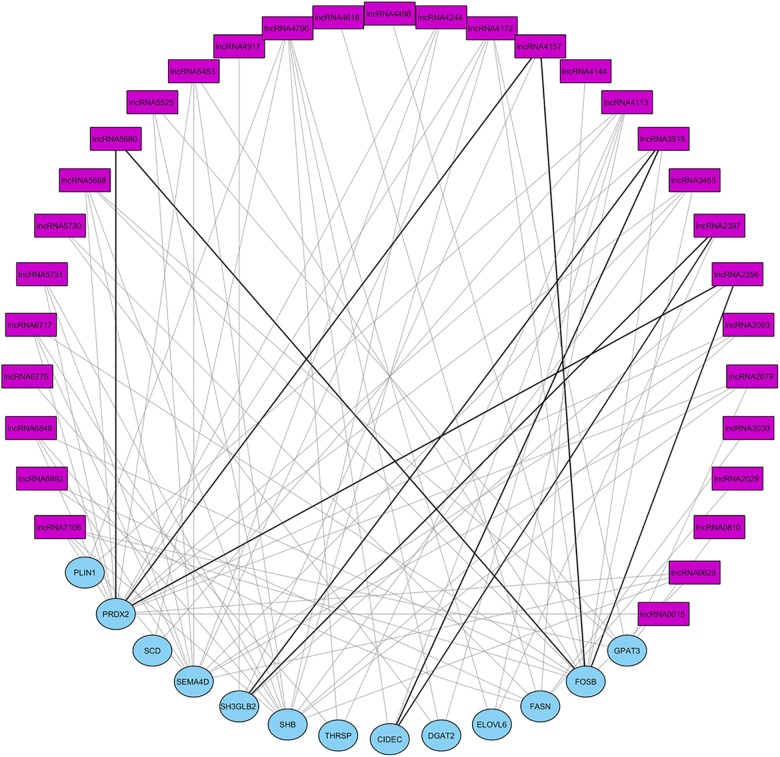
Graphical representation of the correlations DE lncRNA and genes in network 3. Pink square nodes represent lncRNAs and blue circle nodes represent DE genes. Gray lines are positive correlations and black ones are negative correlations. lncRNAXXXX corresponds to lncRNA named in ALDB database as ALDBSSCG000000XXXX.

Co-expression between DE lncRNAs and genes was separately analyzed for one of the top three gene networks. A total of 157 significant correlations were detected between DE lncRNAs expression and genes belonging to gene network 1 (NW1), 121 positive and 36 negative (Supplementary Figure 1). For gene network 2 (NW2) a total of 161 significant correlations were observed, 145 positive and 16 negative (Supplementary Figure 2). Finally, 93 significant correlations were observed for gene network 3 (NW3), 82 positive and 11 negative (Figure 6). Some authors have reported that lncRNAs could act in cis by regulating the expression of their neighboring genes (Zhang et al., 2014). Table 5 shows the position of lncRNAs and correlated genes when they map in the same chromosome. *ALDBSSCG0000002079* could be an activator of the *SEMA4D* gene expression since their expression is positively correlated (ρ = 0.75; *p-*value = 0.005) and they map very close to each other. In contrast, *ALDBSSCG0000005680* and *FOSB* gene expressions are negatively correlated (ρ = -0.82; *p-*value = 0.001), suggesting *ALDBSSCG0000005680* could be involved in the inhibition of *FOSB* gene expression. Another relevant association was identified between *ALDBSSCG0000000629* and *SH2 Domain Containing Adaptor Protein B* (*SHB*) gene which are positively correlated (ρ = 0.882; *p-*value = 1.5 × 10^-4^) and closely located. Although the function of the SHB protein is not well-known, significant associations between two SNPs mapping in this gene and fasting glucose levels (a parameter related with diabetes mellitus type II) has been reported in humans (Welsh et al., 2016).

**Table 5:** Position of lncRNA, closest gene to lncRNA and gene most significantly correlated with lncRNA mapping in the same chromosome.

lncRNA (Position)^1^	Closest gene to lncRNA (Position)^1^/(Position)^2^	Correlated gene (Position)^2^	Correlation
*ALDBSSCG0000000629* (1: 276.85)	*TMEM38B* (1: 276.76)/(1: 246.97)	*SHB* (1: 238.68)	0.747
*ALDBSSCG0000002079* (14: 1.70)	*GADD45G* (14: 1.42)/(14: 1.16)	*SEMA4D* (14: 0.96)	0.882
*ALDBSSCG0000002093* (14: 13.59)	*EXTL3* (14: 13.66)/(14: 12.44)	*SEMA4D* (14: 0.96)	0.903
*ALDBSSCG0000005680* (6: 44.52)	*PLD3* (6: 44.51)/(6: 48.64)	*FOSB* (6: 51.84)	-0.684
*ALDBSSCG0000005453* (6: 64.60)	*PGD* (6: 64.60)/(6: 70.68)	*FOSB* (6: 51.84)	0.920
*ALDBSSCG0000006775* (9: 46.99)	*NXPE4* (9: 47.06)/(9: 42.10)	*THRSP* (9: 12.48)	0.697


*^*1*^Position in Sscrofa10.2; ^*2*^Position in Sscrofa11.1.*

**FIGURE 7:**
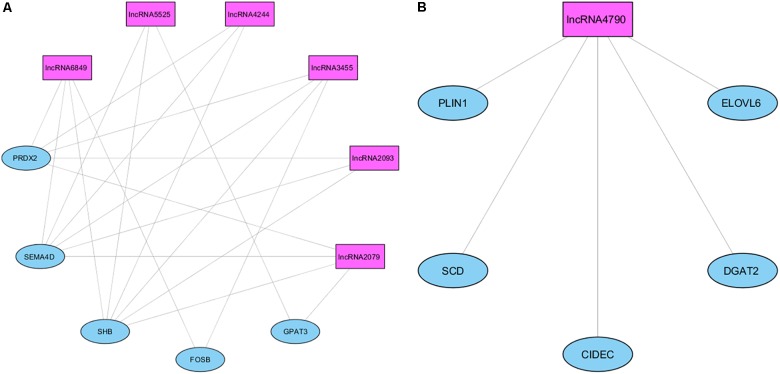
Network of the co-expression between hub lncRNAs and protein-coding genes in the blue **(A)** and turquoise **(B)** modules.

A complementary co-expression network among lncRNAs and protein-coding genes from NW3 was constructed using WGCNA (Langfelder and Horvath, 2008). After clustering of the correlated genes, three modules were obtained (colored blue, gray, and turquoise, see Supplementary Figure 3). Then, a correlation analysis between the modules and the IMF values revealed the turquoise module was strongly correlated with IMF (ρ = 0.76, *p-*value <0.004, Supplementary Figure 4). In contrast, the blue module was inversely correlated with IMF (ρ = -0.57, *p-*value < 0.05). The measures of intramodular connectivity allow detecting six hub lncRNAs (*ALDBSSCG0000002079, ALDBSSCG0000002093, ALDBSSCG0000003455, ALDBSSCG0000004244, ALDBSSCG0000005525*, and *ALDBSSCG0000006849*) in the turquoise module and one hub lncRNA (*ALDBSSCG0000004790*) in the blue module (Figure 7). These seven hub lncRNAs were different to those observed by Zou et al. (2018) in a study of lncRNAs in other pig breeds at earlier developmental stages. The current identification of lncRNAs points out the aforementioned lncRNAs as potential regulators of the DE genes. However, further studies such as methylation analyses, are required to support the regulatory function of these hub lncRNAs.

### Transcription Regulatory Factors Related to Intramuscular Fat

Finally, a regulatory factors study was carried out to analyze other molecular mechanisms underlying the differential expression observed between groups. A total of 1,602 TRF from 1,835 obtained from the transcription factor prediction database showed expression values above 0.5 FPKM. A total of 301 TRFs *z*-scores outside of the lower and upside bounds of bootstrap CIs (Supplementary Table 4) were observed. A total of 186 genes showed extreme *z*-scores for RIF1, 245 for RIF2 and 64 genes both for RIF1 and RIF2. The *Super Elongation Complex Subunit* (*MLLT3*) gene showed the most extreme value for RIF1 (-5.35 SD) and the *CCAAT/Enhancer Binding Protein Gamma* (*CEBPG*) gene showed the most extreme value for RIF2 (-2.52 SD). A total of the 12 identified TRF showed differential expression among H and L groups and six out of this 12 were as well identified as TRF by IPA software (Table 6). However, RIF metrics are particularly interesting for identifying relevant regulatory factors even when they are not DE. From the list of the predicted TRFs both with extreme *z*-scores and recognized by IPA, 44 transcription factors affecting some of the three top gene networks were identified (Supplementary Table 5). For network 1 (NW1) six TRFs were detected (CREB1, CREM, JUNB, MAX, SP1, and STAT3), for network 2 (NW2) 26 TRFs were detected (ATF3, CLOCK, CREB1, CREM, DDIT3, EGR1, FOS, KLF15, KLF2, MAX, MYC, MYOG, NCOA2, NFIL3, NFKB1, POU2F1, PRDM1, RELA, SMAD1, SMAD3, SMAD4, SMARCA1, STAT3, STAT5A, TP63, and VDR) and for network 3 (NW3) 35 TRFs were detected (ATF6, CLOCK, CREB1, CREM, DACH1, EBF1, EGR1, EGR2, ELK1, ELK3, ETS2, FOS, FOSL1, JUNB, KLF15, KLF2, MSC, MYC, MYOG, NCOA1, NCOA2, NCOR1, NFIL3, NFKB1, POU2F1, PRDM1, RELA, SMAD3, SMAD4, SMARCE1, SP1, STAT3, STAT5A, TP63, and ZBTB20).

**Table 6:** Potential regulators affecting gene expression of DE genes between H and L groups, identified by RIFs study and IPA software.

Gene symbol	Gene	RIF1^¥^	RIF2^†^	IPA-regulators *p-*value
*ARID5B*	*ARID Domain-Containing Protein 5B*	-0.10	-2.40	–
*ATF3*	*Activating Transcription Factor 3*	-0.05	-2.23	0.002
*EGR1*	*Early Growth Response 1*	-0.30	-2.18	2.27 × 10^-07^
*EGR2*	*Early Growth Response 2*	-0.23	-2.32	0.015
*EGR3*	*Early Growth Response 3*	-1.70	-2.00	–
*FOS*	*Fos Proto-Oncogene, AP-1 Transcription Factor Subunit*	0.00	-1.87	4.76 × 10^-08^
*JUNB*	*JunB Proto-Oncogene, AP-1 Transcription Factor Subunit*	0.60	-1.34	1.23 × 10^-05^
*KLF11*	*Kruppel Like Factor 11*	-2.30	-0.92	–
*MYC*	*MYC Proto-Oncogene, BHLH Transcription Factor*	-1.70	-1.91	0.003
*MYF6*	*Myogenic Factor 6*	0.10	-2.33	–
*POU3F1*	*POU Class 3 Homeobox 1*	-1.46	-0.81	–
*TRAFD1*	*TRAF-Type Zinc Finger Domain Containing 1*	-0.23	-2.33	–


*^¥^Bootstrap 99% confidence intervals for RIF1 *z*-scores: -1.193/2.352. ^†^Bootstrap 99% confidence intervals for RIF2 *z*-scores: -1.223/1.648.*

Some of these genes have been already mentioned (*EGR1, EGR2, FOS*, and *JUNB*). Among the other genes related with adipogenesis and myogenesis, the *Activating Transcription Factor 3* (*ATF3*) gene is downregulated in the H group (log_2_FC = -1.21, *p-*value = 0.010) and codifies for a protein involved in the positive regulation of cell proliferation and skeletal muscle cell differentiation (Rajan et al., 2012). Differences in the *ATF3* gene expression were also observed among Chinese pig breeds divergent for body weight and IMF (Wang et al., 2015), and it was predicted as a TRF regulating the expression in biceps femori muscle of Iberian purebred and Duroc × Iberian crossbred piglets (Ayuso et al., 2015). The *ARID Domain-Containing Protein 5B* (*ARID5B*) gene codifies for a protein belonging of a DNA binding family proteins. This protein constitutes a histone H3K9Me2 demethylase complex with PHD finger protein 2 and regulates the expression of genes involved in adipogenesis as the *Fat Mass And Obesity-Associated Protein* (*FTO*). A SNP associated with obesity was detected in a long enhancer region of *FTO* gene in human preadipocytes that disrupted the union to ARID5B (Merkestein and Sellayah, 2015). This agrees with the results here presented, since a lower expression of the *ARID5B* gene is observed in the H group (log_2_FC = -1.21, *p-*value = 5 × 10^-5^). *Kruppel Like Factor 11* (*KLF11*) and *Myogenic Factor 6* (*MYF6*) genes codify for proteins with key roles in the regulation of myogenesis, muscle cell differentiation and growth (Kaczynski et al., 2003; Fan et al., 2011). They were also predicted as regulatory factors in LD muscle by Óvilo et al. (2014). Although extreme *z*-scores were predicted for MYC, POU3F1, and TRAFD1, their functional relationship to porcine adipogenesis and muscle development is unknown.

The potential relationships between TRFs and DE genes of the aforementioned networks were explored through the search of transcript factor binding sites (TFBS) with the software Genomatix. This software identifies a number of matches among TRFs and TFBS higher than the expected by chance. TFBS were detected in a mean of 98% of the DE genes (94, 100, 100% for NW 1, 2, 3, respectively). For NW1, CREB, and CREM showed significantly higher number of matches than expected (Benjamini-corrected *p*-value = 0.05). CREB1 and CREM (Benjamini-corrected *p*-value = 0.05), KLF15 and KLF2 (Benjamini-corrected *p*-value = 0.033) and VDR (Benjamini-corrected *p*-value = 0.017) were highlighted for NW2. Lastly, KLF15 and KLF2 (Benjamini-corrected *p*-value = 0.031), CREB1 and CREM (Benjamini-corrected *p*-value = 0.046), EGR1 and EGR2 (Benjamini-corrected *p*-value = 0.046) and ATF6 and MYC (Benjamini-corrected *p*-value = 0.008) and SP1 (Benjamini-corrected *p*-value = 0.008) were overrepresented in NW3. Cyclic AMP response element-binding protein 1 (CREB1) and CAMP Responsive Element Modulator (CREM) are members of the leucine zipper family of DNA binding proteins. These proteins have an essential role during the adipocyte differentiation process (Zhang et al., 2004). Vitamin D receptor (VDR) is activated by Vitamin D and forms a heterodimer with the retinoid X receptor (RXR) and regulates the expression of their target genes. It has been reported that VDR inhibits adipogenesis through repression of the CCAAT enhancer binding protein-alpha and peroxisome proliferator-activated receptor-gamma (PPARγ) (Wood, 2008). Activating Transcription Factor 6 (ATF6) induces the expression of key adipogenic genes. Some authors showed that a reduction of this protein during adipogenesis is related with a decrease of lipid accumulation (Lowe et al., 2012). In addition to this, Sp1 Transcription Factor (SP1) represses the *C/EBPα* gene, which is required for the differentiation from preadipocytes to adipocytes (Tang et al., 1999). As it has been mentioned before, kruppel like factors and early growth factors are involved in myogenesis and muscle development. Therefore, the combined information of extreme RIF *z*-scores, TFBS and biological functions support the regulatory functions of these predicted TRFs and their contribution to the genetic variation of IMF content in the analyzed Iberian pig population.

## Conclusion

In the present study, the LD transcriptomes of 12 Iberian purebred pigs divergent for IMF have been characterized and compared. As a result, a total of 337 DE genes and newly predicted isoforms and 48 lncRNAs were identified. The functional analyses of DE transcripts allowed identifying biochemical and physiological pathways and processes liable of the differences in IMF.

Lipid metabolism genes (*FASN, SCD, ELOVL6, DGAT2, PLIN1, CIDEC*, and *ADIPOQ*) were clearly overexpressed in animals with higher EBV for IMF (H group), whereas genes involved in myogenesis and adipogenesis (*EGR1, EGR2, EGR3, JUNB, FOSB*, and *SEMA4D*) were overexpressed in animals with a lower content in IMF. This agrees with the well-known moderate antagonism between lean meat content and IMF in pigs.

Different analyses toward to identify potential regulators of these DE genes were carried out. In an analysis focused on the network related to lipid metabolism (NW3), seven hub lncRNAs co-expressed with DE genes were identified. The *ALDBSSCG0000002079, ALDBSSCG0000002093, ALDBSSCG0000003455, ALDBSSCG0000004244, ALDBSSCG0000005525*, and *ALDBSSCG0000006849* hub lncRNAs co-expressed with *SEMA4D* and *FOSB*, and are inversely correlated with IMF, and *ALDBSSCG0000004790* is correlated with *SCD, ELOVL6, DGAT2, PLIN1k*, and *CIDEC* and positively correlated with IMF. Moreover, several TRFs potentially responsible of the observed transcription differences were identified. Five of these TRFs have a role in adipogenesis (ARID5B, CREB1, VDR, ATF6, and SP1) and other three take part of myogenesis and development of skeletal muscle (ATF3, KLF11, and MYF6).

Although other functional experiments as methylation pattern analyses and/or promoter studies in cell cultures should be performed to validate the findings showed here, these results provide a set of candidate genes to explain differences in IMF content in Iberian purebred animals. Further steps toward the identification of relevant polymorphisms with extreme allelic frequencies in the divergent groups should be carried out to apply these findings to improve meat quality in Iberian pig.

## Author Contributions

MM participated in animal sampling, gene expression data analyses, and interpretation and drafting of the manuscript. LS and MR participated in experimental design, animal sampling, data interpretation, and drafting of the manuscript. JG-C participated in animal sampling and supervision of the experiments. CC and MF-B participated in animal sampling, RNA isolation, and qPCR analyses. FS-E and FG provided and controlled the animal material and participated in animal sampling.

## Conflict of Interest Statement

The authors declare that the research was conducted in the absence of any commercial or financial relationships that could be construed as a potential conflict of interest.

## Footnotes

1 http://www.bioinformatics.babraham.ac.uk/projects/fastqc/

2 http://www.bioinformatics.babraham.ac.uk/projects/trim_galore/

3 http://res.xaut.edu.cn/aldb/index.jsp

4 http://babelomics.bioinfo.cipf.es/

5 http://www.transcriptionfactor.org/index.cgi?Home

6 http://www.genomatix.de/

7 https://www.ncbi.nlm.nih.gov/tools/primer-blast/
