# Supplementary material for: Identification of Candidate Genes and Regulatory Factors Underlying Intramuscular Fat Content Through Longissimus Dorsi Transcriptome Analyses in Heavy Iberian Pigs
Source: Front Genet. 2018 Dec 4;9:608. doi: 10.3389/fgene.2018.00608 (PMC6288315; doi:10.3389/fgene.2018.00608)
Supplement: Supplementary file 1 [file Data_Sheet_1.docx]

Supplementary Material

Identification of candidate genes and regulatory factors underlying intramuscular fat content through longissimus dorsi transcriptome analyses in heavy Iberian pigs.

M. Muñoz^*^, J.M. García-Casco, C. Caraballo, M.A. Fernández-Barroso, F. Sánchez-Esquiliche, F. Gómez, M.C. Rodríguez & L. Silió.

*** Correspondence:** María Muñoz: mariamm@inia.es

# Supplementary Data

# Supplementary Figures and Tables

## Supplementary Figures


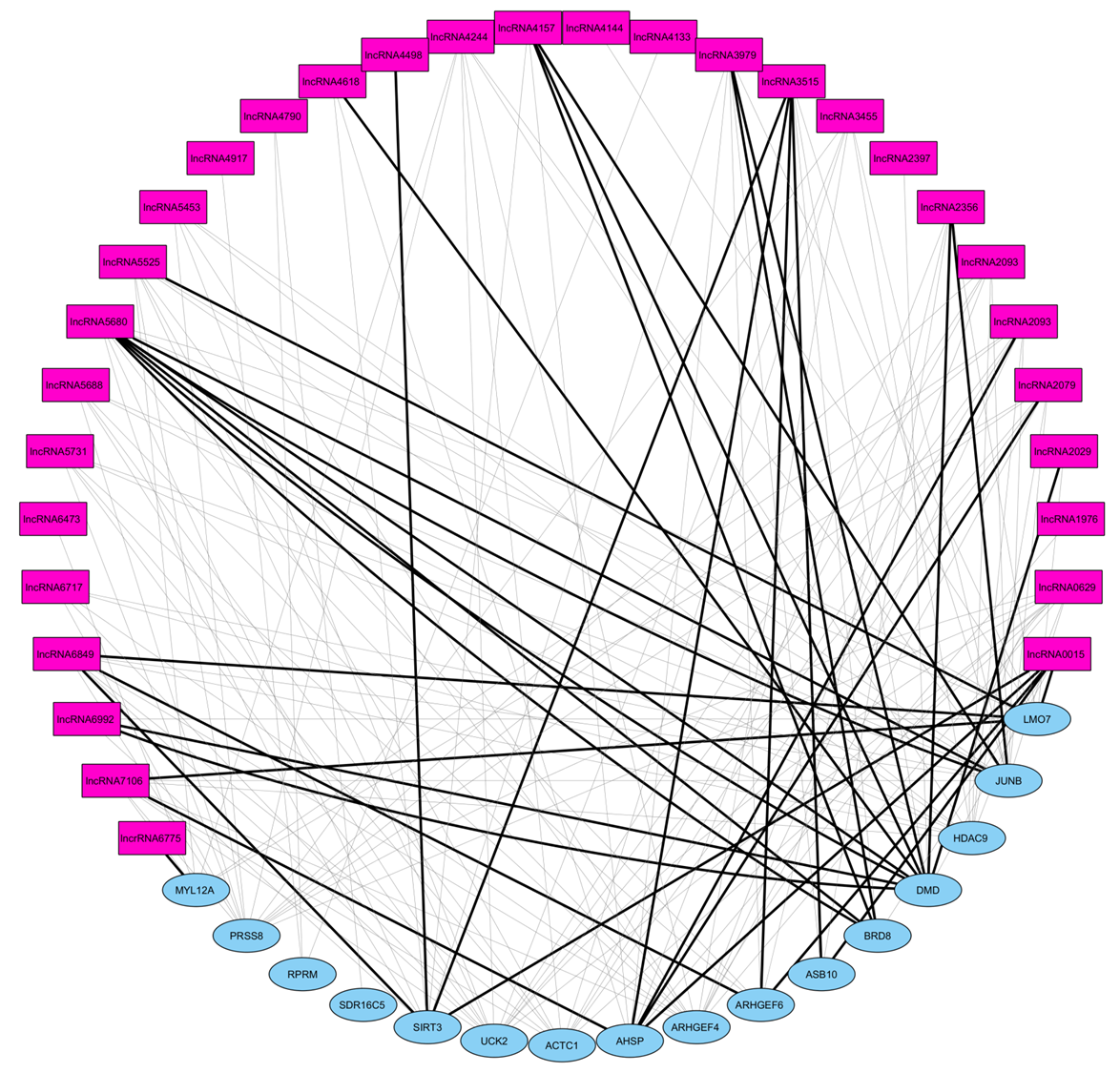


**Supplementary Figure 1.** Graphical representation of the correlations DE lncRNA and genes in network 1. Pink square nodes represent lncRNAs and blue circle nodes represent DE genes. Grey lines are positive correlations and black ones are negative correlations. lncRNAXXXX corresponds to lncRNA named in ALDB database as ALDBSSCG000000XXXX.


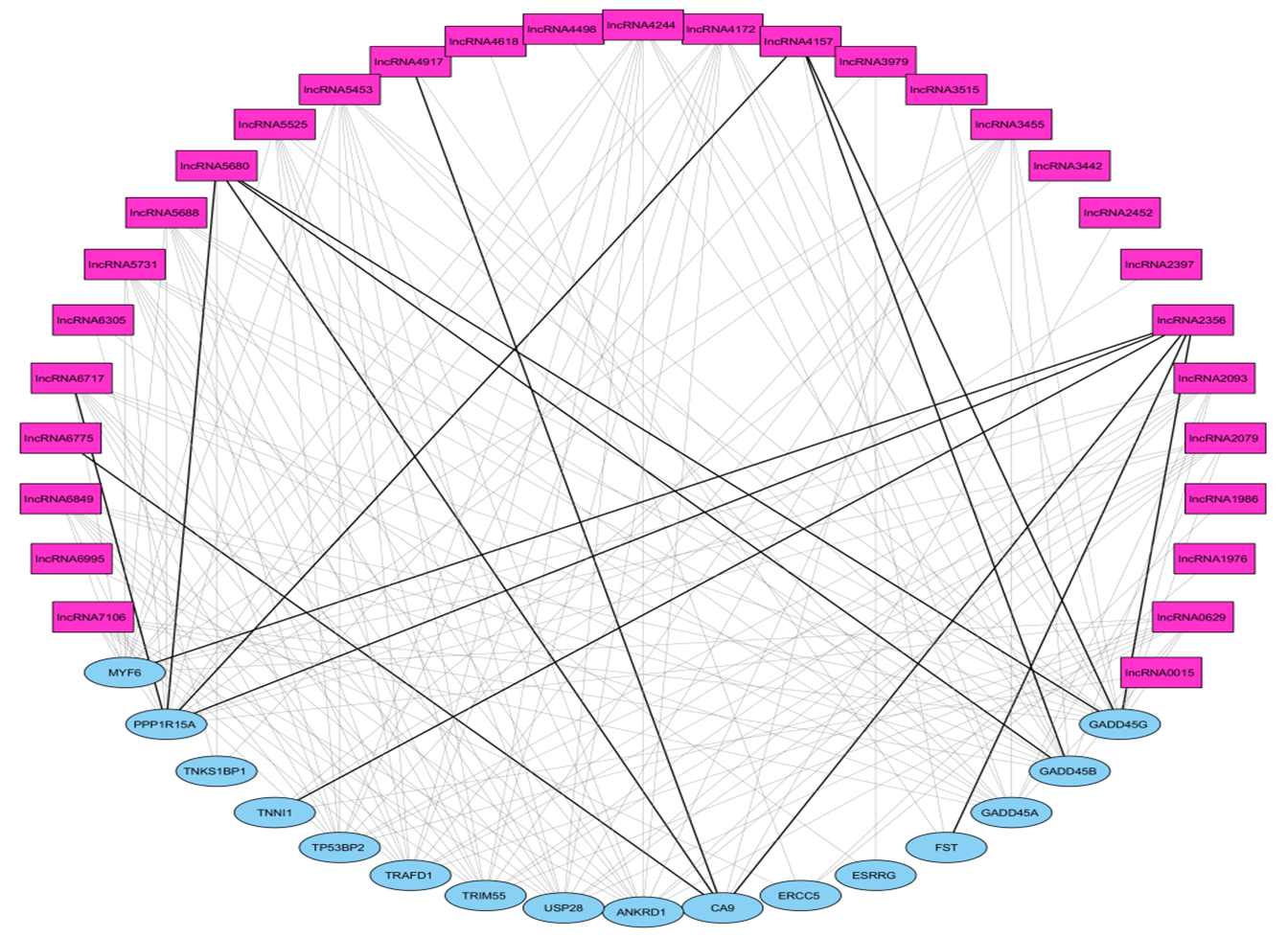


**Supplementary Figure 2.** Graphical representation of the correlations DE lncRNA and genes in network 2. Pink square nodes represent lncRNAs and blue circle nodes represent DE genes. Grey lines are positive correlations and black ones are negative correlations. lncRNAXXXX corresponds to lncRNA named in ALDB database as ALDBSSCG000000XXXX.

**
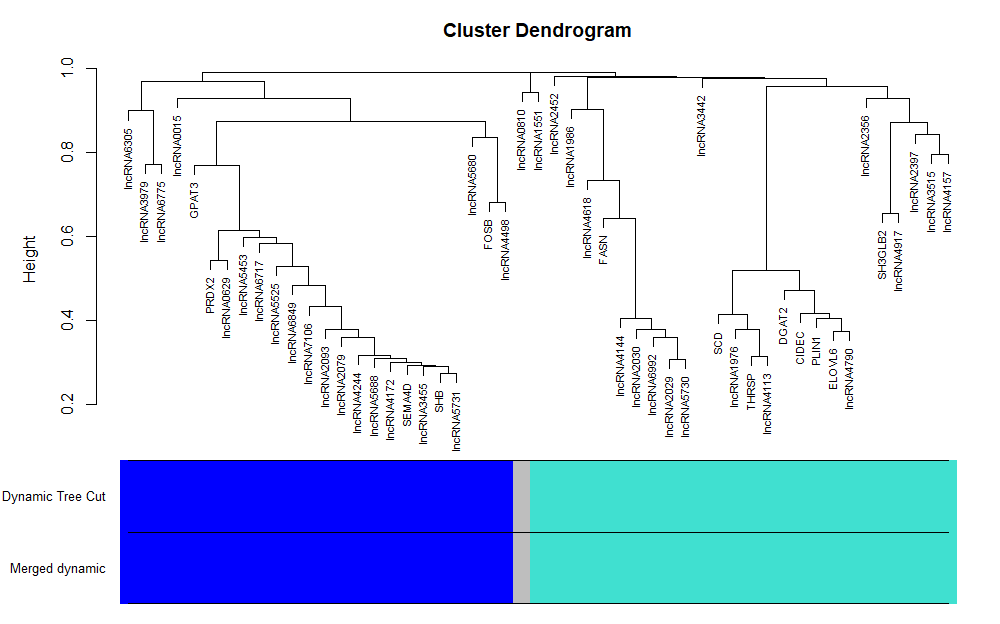
**

**Supplementary Figure 3.** Gene dendrogram based on the consensus topological overlap matrix and gene clustering using hierarchical clustering and dynamic tree cutting. The identified modules are indicated in the color of the row under the dendrogram.


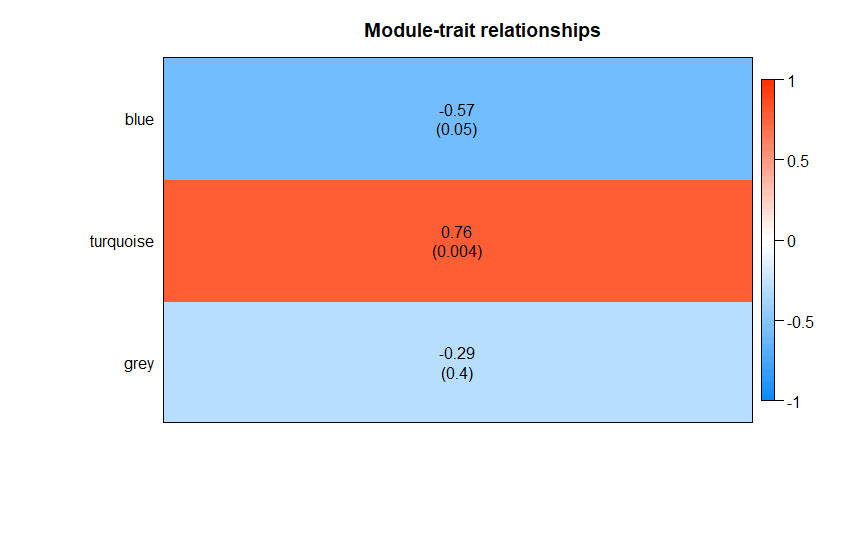


**Supplementary Figure 4.** Module-stage correlations and their corresponding *p*-values. “blue”, “turquoise” and “grey” correspond to different modules. Each module contains the correlation estimate and *p*-value between brackets. Modules are color-codes by the correlation according to the color legend on the right.

## Supplementary Tables

**Supplementary Table 1**. Primer name, sequences and size of genes selected for qPCR validation.

| **Name** | **Sequence 5’3’** | **Primer Size** |
| --- | --- | --- |
| ITGB6F | TGGATGACGACCTCAACACG | 20 |
| ITGB6R | TACTACTGCAAGGGTTGGCG | 20 |
| DCAF5F | GGTTGCTGACTGGATCCCAA | 20 |
| DCAF5R | GCAGTGAGGATGTCCTAGCC | 20 |
| CNOT10F | GAAGCCCTCATCTCTCTGGAC | 21 |
| CNOT10R | CCCTTTACAACCTTGCCCTT | 20 |
| GPHNF | GTGAACAGCCGACTCAGACA | 20 |
| GPHNR | TGCCATCATCGGATTCCCTG | 20 |
| SCDF | AATGCCACCTGGCTGGTAAA | 20 |
| SCDR | GTGGAAGCCCTCACCCACAG | 20 |
| FASNF | AGTAAGCCCAAGTACAGCGG | 20 |
| FASNR | CTCACGGAGGAGAAGATCACG | 21 |
| PFKFB3F | ATGAGAGTCCGCAAGCAGTG | 20 |
| PFKFB3R | ATGTTTGAGGCCACGACTGT | 20 |
| EGR1F | GAGGGCAGCGGCGGTAACAG | 20 |
| EGR1R | GGGAAAAGACTCTGCGGTCAGGTG | 24 |
| SPP1F | GCAGTGATAGCCTTCTGTCT | 20 |
| SPP1R | TGTGGCGCTAGGAAAGTCTG | 20 |
| DNAJA1F | ATTGAAAAAGGCTTACAGGAAACT | 24 |
| DNAJA1R | GGGGGAGCCAAAACCACCAC | 20 |
| FOSF | ATCCCAACGGTGACTGCTATCTCG | 24 |
| FOSR | TGCCCCTTCTGCCAATGCTCT | 21 |
| ADIPOQF | CCTTCACCTCCTTCATGCTCTCCT | 24 |
| ADIPOQR | GGCGAGAAGGGTGAGAAAGGAGAT | 23 |
| ATF3F | ACCCCTCGAGATGTCAGTCAC | 21 |
| ATF3R | CTCCTCAATTTGGGCCTTCAGTTC | 24 |
| ELOVL6F | AGAACACGTAGCGACTCCGAAGAT | 24 |
| ELOVL6R | GACATGCCGACCGCCAAAGATAA | 23 |
| PLIN1F | CCCCCTGGTGGCGTCTGTAT | 20 |
| PLIN1R | ACTGGAGGGCCGGTATCTTTTCT | 23 |

**Supplementary Table 2.** Total number of reads, filtered reads, and percentage of mapped reads per sample.

| **Type** | **Total Reads, n** | **Filtered Reads, n** | **Mapped results, %** |
| --- | --- | --- | --- |
| H1 | 139,583,278 | 139,021,798 | 95.4 |
| H2 | 120,144,210 | 119,522,032 | 94.3 |
| H3 | 137,201,242 | 136,625,832 | 95.1 |
| H4 | 161,747,406 | 161,059,450 | 95.0 |
| H5 | 105,988,344 | 105,559,330 | 94.5 |
| H6 | 154,892,848 | 154,288,790 | 95.3 |
| L1 | 130,967,634 | 130,430,178 | 94.7 |
| L2 | 141,699,342 | 141,206,490 | 94.7 |
| L3 | 117,605,580 | 117,215,922 | 94.9 |
| L4 | 142,698,630 | 142,167,082 | 95.5 |
| L5 | 118,793,246 | 118,373,744 | 95.3 |
| L6 | 158,290,780 | 157,699,648 | 94.7 |

H1-H6: Individuals with high breeding value for %IMF; L1-L6: Individuals with low breeding value for %IMF.

**Supplementary Table 3**. Overrepresented GO terms related of the differentially expressed genes between H and L groups using FatiGO.

| Term | Genes | Adjusted *p-*value |
| --- | --- | --- |
| Response to camp(GO:0051591) | *EGR1,EGR3,EGR2,JUNB,FOS,FOSB,DUSP1,ADIPOQ* | 1.30x10^-9^ |
| Response to alcohol(GO:0097305) | *EGR1,CTGF,JUNB,SPP1,FOS,CA9,FOSB,DUSP1,ACTC1,ADIPOQ* | 2.40x10^-9^ |
| Response to organophosphorus(GO:0046683) | *EGR1,EGR3,EGR2,JUNB,FOS,FOSB,DUSP1,ADIPOQ* | 8.10x10^-9^ |
| Response to purine-containing compound(GO:0014074) | *EGR1,EGR3,EGR2,JUNB,FOS,FOSB,DUSP1,ADIPOQ* | 1.23x10^-8^ |
| Skeletal muscle tissue development(GO:0007519) | *EGR1,EGR2,MYF6,ATF3,FOS,ANKRD1,DMD,HDAC9* | 3.19x10^-8^ |
| Skeletal muscle organ development(GO:0060538) | *EGR1,EGR2,MYF6,ATF3,FOS,ANKRD1,DMD,HDAC9* | 4.64x10^-8^ |
| Skeletal muscle cell differentiation(GO:0035914) | *EGR1,EGR2,MYF6,ATF3,FOS,ANKRD1* | 4.76x10^-8^ |
| Response to ketone(GO:1901654) | *JUNB,FOS,CA9,FOSB,ADCY6,DUSP1* | 1.53x10^-6^ |
| Response to corticosteroid(GO:0031960) | *CTGF,JUNB,FOS,FOSB,DUSP1,ADIPOQ* | 3.39x10^-6^ |
| Response to mineralocorticoid(GO:0051385) | *CTGF,JUNB,FOS,FOSB* | 7.69x10^-6^ |
| Negative regulation of DNA biosynthetic process(GO:2000279) | *ANKRD1,DUSP1,ADIPOQ* | 3.86x10^-5^ |
| Muscle organ morphogenesis(GO:0048644) | *MYF6,ANKRD1,ACTC1,ARID5B,TNNI1* | 3.86x10^-5^ |
| Response to oxygen levels(GO:0070482) | *EGR1,CTGF,NR4A2,CA9,ANKRD1,UCK2,ADIPOQ* | 3.93x10^-5^ |
| Response to mechanical stimulus(GO:0009612) | *EGR1,JUNB,FOS,ANKRD1,FOSB,GADD45A* | 4.17x10^-5^ |
| Response to glucocorticoid(GO:0051384) | *JUNB,FOS,FOSB,DUSP1,ADIPOQ* | 6.64x10^-5^ |
| Activation of MAPKKK activity(GO:0000185) | *GADD45G,GADD45B,GADD45A* | 1.19x10^-4^ |
| Regulation of DNA biosynthetic process(GO:2000278) | *ANKRD1,MYC,DUSP1,ADIPOQ* | 1.38x10^-4^ |
| Response to fatty acid(GO:0070542) | *CTGF,ADCY6,ADIPOQ,DGAT2* | 1.38x10^-4^ |
| Positive regulation of p38mapk cascade(GO:1900745) | *GADD45G,GADD45B,GADD45A* | 1.38x10^-4^ |
| Connective tissue development(GO:0061448) | *EGR1,CTGF,TNMD,CYR61,ARID5B,DGAT2* | 1.38x10^-4^ |
| Cellular response to camp(GO:0071320) | *EGR1,EGR3,EGR2,ADIPOQ* | 1.93x10^-4^ |
| Response to corticosterone(GO:0051412) | *JUNB,FOS,FOSB* | 2.15x10^-4^ |
| Response to acid chemical(GO:0001101) | *EGR1,CTGF,ADCY6,DUSP1,ADIPOQ,DGAT2* | 2.15x10^-4^ |
| Negative regulation of protein kinase activity(GO:0006469) | *PPP1R1B,DUSP1,ADIPOQ,GADD45G,GADD45B,GADD45A* | 2.44x10^-4^ |
| DNA biosynthetic process(GO:0071897) | *CTGF,ANKRD1,MYC,DUSP1,ADIPOQ* | 2.59x10^-4^ |
| Negative regulation of kinase activity(GO:0033673) | *PPP1R1B,DUSP1,ADIPOQ,GADD45G,GADD45B,GADD45A* | 2.87x10^-4^ |
| Regulation of p38mapk cascade(GO:1900744) | *GADD45G,GADD45B,GADD45A* | 2.87x10^-4^ |
| Cellular response to drug(GO:0035690) | *EGR1,ANKRD1,MYC,ADIPOQ* | 2.87x10^-4^ |
| Response to decreased oxygen levels(GO:0036293) | *EGR1,CTGF,NR4A2,CA9,ANKRD1,ADIPOQ* | 2.87x10^-4^ |
| Triglyceride metabolic process(GO:0006641) | *ELOVL6,THRSP,FASN,PLIN1,DGAT2* | 3.75x10^-4^ |
| P38mapk cascade(GO:0038066) | *GADD45G,GADD45B,GADD45A* | 3.75x10^-4^ |
| Striated muscle contraction(GO:0006941) | *CTGF,SCN3B,DMD,ACTC1,TNNI1* | 3.75x10^-4^ |
| Cellular response to gonadotropin stimulus(GO:0071371) | *EGR1,EGR3,EGR2* | 3.75x10^-4^ |
| Response to amphetamine(GO:0001975) | *EGR1,NR4A2,PPP1R1B* | 3.75x10^-4^ |
| Striated muscle cell development(GO:0055002) | *FHOD3,ANKRD1,DMD,HDAC9,ACTC1* | 3.99x10^-4^ |
| Neutral lipid metabolic process(GO:0006638) | *ELOVL6,THRSP,FASN,PLIN1,DGAT2* | 4.27x10^-4^ |
| Acylglycerol metabolic process(GO:0006639) | *ELOVL6,THRSP,FASN,PLIN1,DGAT2* | 4.27x10^-4^ |
| Positive regulation of JNK cascade(GO:0046330) | *CTGF,GADD45G,GADD45B,GADD45A* | 4.42x10^-4^ |
| Muscle cell development(GO:0055001) | *FHOD3,ANKRD1,DMD,HDAC9,ACTC1* | 4.91x10^-4^ |
| Muscle tissue morphogenesis(GO:0060415) | *MYF6,ANKRD1,ACTC1,TNNI1* | 4.91x10^-4^ |
| Response to ionizing radiation(GO:0010212) | *USP28,EGR1,MYC,TP53BP2,GADD45A* | 7.01x10^-4^ |
| Response to amine(GO:0014075) | *EGR1,NR4A2,PPP1R1B* | 7.47x10^-4^ |
| Peripheral nervous system development(GO:0007422) | *EGR3,EGR2,POU3F1,DMD* | 7.47x10^-4^ |
| Regulation of metanephros development(GO:0072215) | *EGR1,MYC,ADIPOQ* | 7.74x10^-4^ |
| Response to gonadotropin(GO:0034698) | *EGR1,EGR3,EGR2* | 8.12x10^-4^ |
| Multi-multicellular organism process(GO:0044706) | *JUNB,SPP1,PPP1R1B,FOS,FOSB* | 8.28x10^-4^ |
| Fat pad development(GO:0060613) | *ARID5B,DGAT2* | 8.77x10^-4^ |
| Positive regulation of stress-activated MAPK cascade(GO:0032874) | *CTGF,GADD45G,GADD45B,GADD45A* | 8.92x10^-4^ |
| Positive regulation of stress-activated protein kinase signaling cascade(GO:0070304) | *CTGF,GADD45G,GADD45B,GADD45A* | 9.19x10^-4^ |
| Response to progesterone(GO:0032570) | *JUNB,FOS,FOSB* | 9.46x10^-4^ |
| Heart contraction(GO:0060047) | *CTGF,HBEGF,SCN3B,DMD,ACTC1* | 9.46x10^-4^ |
| Heart process(GO:0003015) | *CTGF,HBEGF,SCN3B,DMD,ACTC1* | 9.67x10^-4^ |
| Regulation of stress-activated MAPK cascade(GO:0032872) | *CTGF,MYC,GADD45G,GADD45B,GADD45A* | 1.13x10^-3^ |
| Regulation of stress-activated protein kinase signaling cascade(GO:0070302) | *CTGF,MYC,GADD45G,GADD45B,GADD45A* | 1.13x10^-3^ |
| Cardiac muscle contraction(GO:0060048) | *CTGF,SCN3B,DMD,ACTC1* | 1.42x10^-3^ |
| Triglyceride biosynthetic process(GO:0019432) | *ELOVL6,THRSP,FASN,DGAT2* | 1.49x10^-3^ |
| Response to monoamine(GO:0071867) | *EGR1,ADCY6,ADIPOQ* | 1.53x10^-3^ |
| Response to catecholamine(GO:0071869) | *EGR1,ADCY6,ADIPOQ* | 1.53x10^-3^ |
| Regulation of striated muscle contraction(GO:0006942) | *CTGF,SCN3B,DMD,TNNI1* | 1.53x10^-3^ |
| Striated muscle cell differentiation(GO:0051146) | *FHOD3,ANKRD1,DMD,HDAC9,ACTC1* | 1.53x10^-3^ |
| Neutral lipid biosynthetic process(GO:0046460) | *ELOVL6,THRSP,FASN,DGAT2* | 1.53x10^-3^ |
| Acylglycerol biosynthetic process(GO:0046463) | *ELOVL6,THRSP,FASN,DGAT2* | 1.53x10^-3^ |
| JNK cascade(GO:0007254) | *ARHGEF6,CTGF,GADD45G,GADD45B,GADD45A* | 1.61x10^-3^ |
| Positive regulation of cell proliferation involved in kidney development(GO:1901724) | *EGR1,MYC* | 1.80x10^-3^ |
| Long-chain fatty-acyl-coa metabolic process(GO:0035336) | *ELOVL6,FASN,DGAT2* | 1.83x10^-3^ |
| Response to hypoxia(GO:0001666) | *EGR1,NR4A2,CA9,ANKRD1,ADIPOQ* | 2.33x10^-3^ |
| Transforming growth factor beta receptor signaling pathway(GO:0007179) ^-^ | *JUNB,PEG10,FOS,MYC,PPP1R15A* | 2.44x10^-3^ |
| Placenta development(GO:0001890) | *JUNB,SPP1,PEG10,CYR61* | 2.44x10^-3^ |
| Lipid particle(GO:0005811) | *CIDEC,PLIN1,DGAT2* | 2.62x10^-3^ |
| Peptidyl-lysine deacetylation(GO:0034983) | *SIRT3,HDAC9* | 2.68x10^-3^ |
| Fatty-acyl-coa metabolic process(GO:0035337) | *ELOVL6,FASN,DGAT2* | 2.84x10^-3^ |
| Regulation of metanephric glomerulus development(GO:0072298) | *EGR1,ADIPOQ* | 3.19x10^-3^ |
| Response to calcium ion(GO:0051592) | *JUNB,FOS,FOSB,DUSP1* | 3.36x10^-3^ |
| Glial cell differentiation(GO:0010001) | *EGR1,EGR2,POU3F1,DMD* | 3.64x10^-3^ |
| Negative regulation of neuron projection development(GO:0010977) | *SPP1,ADCY6,DGUOK* | 3.87x10^-3^ |
| Cellular response to oxygen levels(GO:0071453) | *EGR1,CA9,ANKRD1,UCK2* | 4.31x10^-3^ |
| Platelet-derived growth factor receptor signaling pathway(GO:0048008) | *CSRNP1,ARID5B,ADIPOQ* | 4.45x10^-3^ |
| Female pregnancy(GO:0007565) | *JUNB,SPP1,FOS,FOSB* | 5.21x10^-3^ |
| Cellular metabolic compound salvage(GO:0043094) | *AMPD3,UCK2,DGUOK* | 5.40x10^-3^ |
| Regulation of kidney development(GO:0090183) | *EGR1,MYC,ADIPOQ* | 5.42x10^-3^ |
| Cranial nerve structural organization(GO:0021604) | *EGR2,DMD* | 5.42x10^-3^ |
| Protein sumoylation(GO:0016925) | *EGR1,EGR2,RASD2* | 5.51x10^-3^ |
| Sodium ion transmembrane transport(GO:0035725) | *SCN3B,SCNN1D,SLC4A7,DMD* | 5.69x10^-3^ |
| Actin-myosin filament sliding(GO:0033275) | *DMD,ACTC1,TNNI1* | 5.80x10^-3^ |
| Muscle filament sliding(GO:0030049) | *DMD,ACTC1,TNNI1* | 5.80x10^-3^ |
| Gliogenesis(GO:0042063) | *EGR1,EGR2,POU3F1,DMD* | 5.87x10^-3^ |
| Myofibril assembly(GO:0030239) | *FHOD3,ANKRD1,ACTC1* | 5.87x10^-3^ |
| Epithelial cell development(GO:0002064) | *SLC4A7,DMD,TNMD,ADIPOQ* | 5.89x10^-3^ |
| Cellular response to calcium ion(GO:0071277) | *JUNB,FOS,FOSB* | 5.89x10^-3^ |
| Cardiac muscle tissue development(GO:0048738) | *FHOD3,ANKRD1,ACTC1,TNNI1* | 6.13x10^-3^ |
| Cell proliferation involved in metanephros development(GO:0072203) | *EGR1,MYC* | 6.20x10^-3^ |
| Cardiac muscle tissue morphogenesis(GO:0055008) | *ANKRD1,ACTC1,TNNI1* | 6.50x10^-3^ |
| Regulation of muscle contraction(GO:0006937) | *CTGF,SCN3B,DMD,TNNI1* | 6.57x10^-3^ |
| Lamellipodium assembly(GO:0030032) | *ARHGEF4,ARHGEF6,RHOD* | 6.57x10^-3^ |
| Regulation of cell-substrate adhesion(GO:0010810) | *RHOD,SPP1,DMD,CYR61* | 6.58x10^-3^ |
| Regulation of JNK cascade(GO:0046328) | *CTGF,GADD45G,GADD45B,GADD45A* | 6.93x10^-3^ |
| Learning or memory(GO:0007611) | *EGR1,EGR2,PPP1R1B,FOS* | 7.19x10^-3^ |
| Negative regulation of protein binding(GO:0032091) | *SLPI,NES,ADIPOQ* | 7.31x10^-3^ |
| Reactive oxygen species metabolic process(GO:0072593) | *PRDX2,CTGF,CYR61,GADD45A* | 7.78x10^-3^ |
| Regulation of cell proliferation involved in kidney development(GO:1901722) | *EGR1,MYC* | 7.78x10^-3^ |
| Regulation of glomerulus development(GO:0090192) | *EGR1,ADIPOQ* | 7.78x10^-3^ |
| Lamellipodium organization(GO:0097581) | *ARHGEF4,ARHGEF6,RHOD* | 7.94x10^-3^ |
| Cellular response to radiation(GO:0071478) | *USP28,EGR1,MYC,GADD45A* | 7.98x10^-3^ |
| Regulation of heart contraction(GO:0008016) | *CTGF,HBEGF,SCN3B,DMD* | 8.18x10^-3^ |
| Regulation of sodium ion transport(GO:0002028) | *PRSS8,SCN3B,DMD* | 8.99x10^-3^ |
| Myeloid leukocyte differentiation(GO:0002573) | *JUNB,FOS,MYC,ADIPOQ* | 8.99x10^-3^ |
| Post-embryonic development(GO:0009791) | *NR4A2,CSRNP1,ARID5B* | 8.99x10^-3^ |
| Positive regulation of cell adhesion(GO:0045785) | *RHOD,SPP1,DMD,CYR61* | 9.10x10^-3^ |
| Cellular response to mechanical stimulus(GO:0071260) | *EGR1,ANKRD1,GADD45A* | 0.01 |
| Response to ethanol(GO:0045471) | *EGR1,ACTC1,ADIPOQ* | 0.01 |
| Regulation of neuron apoptotic process(GO:0043523) | *PRDX2,EGR1,NR4A2,NES* | 0.01 |
| Anatomical structure arrangement(GO:0048532) | *EGR2,DMD* | 0.01 |
| Actin-mediated cell contraction(GO:0070252) | *DMD,ACTC1,TNNI1* | 0.01 |
| Heart morphogenesis(GO:0003007) | *ANKRD1,CYR61,ACTC1,TNNI1* | 0.01 |
| Regulation of muscle system process(GO:0090257) | *CTGF,SCN3B,DMD,TNNI1* | 0.01 |
| Metanephros development(GO:0001656) | *EGR1,MYC,ADIPOQ* | 0.01 |
| Labyrinthine layer blood vessel development(GO:0060716) | *JUNB,CYR61* | 0.01 |
| Metanephric glomerulus development(GO:0072224) | *EGR1,ADIPOQ* | 0.01 |
| Regulation of ERK1 and ERK2 cascade(GO:0070372) | *CTGF,DUSP1,CYR61,ADIPOQ* | 0.01 |
| Action potential(GO:0001508) | *EGR2,POU3F1,SCN3B,DMD* | 0.01 |
| Response to testosterone(GO:0033574) | *CA9,DUSP1* | 0.01 |
| Actomyosin structure organization(GO:0031032) | *FHOD3,ANKRD1,ACTC1* | 0.01 |
| Cognition(GO:0050890) | *EGR1,EGR2,PPP1R1B,FOS* | 0.01 |
| Cell proliferation involved in kidney development(GO:0072111) | *EGR1,MYC* | 0.01 |
| Regulation of transmembrane receptor protein serine/threonine kinase signaling pathway(GO:0090092) | *PEG10,FST,PPP1R15A,CYR61* | 0.01 |
| Osteoblast differentiation(GO:0001649) | *JUNB,FASN,SPP1,CYR61* | 0.01 |
| ERK1 and ERK2 cascade(GO:0070371) | *CTGF,DUSP1,CYR61,ADIPOQ* | 0.02 |
| Acyl-coa metabolic process(GO:0006637) | *ELOVL6,FASN,DGAT2* | 0.02 |
| Response to isoquinoline alkaloid(GO:0014072) | *EGR1,FOSB* | 0.02 |
| Thioester metabolic process(GO:0035383) | *ELOVL6,FASN,DGAT2* | 0.02 |
| Neuron apoptotic process(GO:0051402) | *PRDX2,EGR1,NR4A2,NES* | 0.02 |
| Regulation of cardiac muscle contraction(GO:0055117) | *CTGF,SCN3B,DMD* | 0.02 |
| Nucleobase metabolic process(GO:0009112) | *AMPD3,UCK2,DGUOK* | 0.02 |
| Regulation of leukocyte differentiation(GO:1902105) | *EGR3,FOS,MYC,ADIPOQ* | 0.02 |
| Positive regulation of cell-substrate adhesion(GO:0010811) | *SPP1,DMD,CYR61* | 0.02 |
| Cranial nerve morphogenesis(GO:0021602) | *EGR2,DMD* | 0.02 |
| Adipose tissue development(GO:0060612) | *ARID5B,DGAT2* | 0.02 |
| Regulation of synaptic transmission(GO:0050804) | *EGR1,EGR2,KCNC4,ADIPOQ* | 0.02 |
| Actin filament-based movement(GO:0030048) | *DMD,ACTC1,TNNI1* | 0.02 |
| Decidualization(GO:0046697) | *JUNB,SPP1* | 0.02 |
| Low-density lipoprotein particle clearance(GO:0034383) | *ADIPOQ,DGAT2* | 0.02 |
| Kidney development(GO:0001822) | *EGR1,MYC,ARID5B,ADIPOQ* | 0.02 |
| Regulation of neuron death(GO:1901214) | *PRDX2,EGR1,NR4A2,NES* | 0.02 |
| Cardiac myofibril assembly(GO:0055003) | *FHOD3,ACTC1* | 0.02 |
| Epidermis development(GO:0008544) | *POU3F1,CTGF,FST,SLC4A7* | 0.02 |
| Membrane hyperpolarization(GO:0060081) | *IGSF9B,ADIPOQ* | 0.02 |
| Negative regulation of cell projection organization(GO:0031345) | *SPP1,ADCY6,DGUOK* | 0.02 |
| Negative regulation of DNA metabolic process(GO:0051053) | *ANKRD1,DUSP1,ADIPOQ* | 0.02 |
| Negative regulation of binding(GO:0051100) | *SLPI,NES,ADIPOQ* | 0.02 |
| Rhythmic process(GO:0048511) | *EGR1,EGR3,EGR2,ADIPOQ* | 0.02 |
| Placenta blood vessel development(GO:0060674) | *JUNB,CYR61* | 0.02 |
| Regulation of myeloid leukocyte differentiation(GO:0002761) | *FOS,MYC,ADIPOQ* | 0.02 |
| Response to glucose(GO:0009749) | *EGR1,CTGF,ADIPOQ* | 0.02 |
| Nucleotide salvage(GO:0043173) | *AMPD3,UCK2* | 0.02 |
| Hindbrain development(GO:0030902) | *EGR2,PTPRS,SLC4A7* | 0.02 |
| Response to metal ion(GO:0010038) | *JUNB,FOS,FOSB,DUSP1* | 0.02 |
| Regulation of protein sumoylation(GO:0033233) | *EGR1,RASD2* | 0.02 |
| Retinol metabolic process(GO:0042572) | *SDR16C5,DGAT2* | 0.02 |
| Positive regulation of sodium ion transport(GO:0010765) | *PRSS8,SCN3B* | 0.02 |
| Renal system development(GO:0072001) | *EGR1,MYC,ARID5B,ADIPOQ* | 0.02 |
| Cellular response to metal ion(GO:0071248) | *JUNB,FOS,FOSB* | 0.02 |
| Response to hexose(GO:0009746) | *EGR1,CTGF,ADIPOQ* | 0.02 |
| Purine-containing compound salvage(GO:0043101) | *AMPD3,DGUOK* | 0.02 |
| Response to monosaccharide(GO:0034284) | *EGR1,CTGF,ADIPOQ* | 0.03 |
| Maternal placenta development(GO:0001893) | *JUNB,SPP1* | 0.03 |
| Histone H3 deacetylation(GO:0070932) | *SIRT3,HDAC9* | 0.03 |
| Negative regulation of transmembrane receptor protein serine/threonine kinase signaling pathway(GO:0090101) | *PEG10,FST,PPP1R15A* | 0.03 |
| Cellular response to inorganic substance(GO:0071241) | *JUNB,FOS,FOSB* | 0.03 |
| Cellular response to fatty acid(GO:0071398) | *ADCY6,DGAT2* | 0.03 |
| Long-chain fatty-acyl-coa biosynthetic process(GO:0035338) | *ELOVL6,FASN* | 0.03 |
| Negative regulation of neuron apoptotic process(GO:0043524) | *PRDX2,NR4A2,NES* | 0.03 |
| Nucleoside monophosphate biosynthetic process(GO:0009124) | *AMPD3,UCK2,DGUOK* | 0.03 |
| Cellular response to hypoxia(GO:0071456) | *EGR1,CA9,ANKRD1* | 0.03 |
| Fatty-acyl-coa biosynthetic process(GO:0046949) | *ELOVL6,FASN* | 0.03 |
| Cellular response to decreased oxygen levels(GO:0036294) | *EGR1,CA9,ANKRD1* | 0.03 |
| Response to carbohydrate(GO:0009743) | *EGR1,CTGF,ADIPOQ* | 0.04 |
| BMP signaling pathway(GO:0030509) | *EGR1,FST,CYR61* | 0.04 |
| Cellular response to monoamine stimulus(GO:0071868) | *ADCY6,ADIPOQ* | 0.04 |
| Cellular response to catecholamine stimulus(GO:0071870) | *ADCY6,ADIPOQ* | 0.04 |
| Schwann cell differentiation(GO:0014037) | *EGR2,POU3F1* | 0.04 |
| Epiboly involved in wound healing(GO:0090505) | *HBEGF,CYR61* | 0.04 |
| Wound healing, spreading of cells(GO:0044319) | *HBEGF,CYR61* | 0.04 |
| Face morphogenesis(GO:0060325) | *CSRNP1,ARID5B* | 0.04 |
| Cellular response to acid chemical(GO:0071229) | *EGR1,ADCY6,DGAT2* | 0.04 |
| Epiboly(GO:0090504) | *HBEGF,CYR61* | 0.04 |
| Osteoblast proliferation(GO:0033687) | *JUNB,CYR61* | 0.04 |
| Cranial nerve development(GO:0021545) | *EGR2,DMD* | 0.04 |
| Response to axon injury(GO:0048678) | *SPP1,UCK2* | 0.04 |
| Response to toxic substance(GO:0009636) | *EGR1,NR4A2,FOS* | 0.04 |
| Cell migration involved in sprouting angiogenesis(GO:0002042) | *EGR3,HDAC9* | 0.04 |
| Regulation of protein binding(GO:0043393) | *SLPI,NES,ADIPOQ* | 0.04 |
| Regulation of sodium ion transmembrane transporter activity(GO:2000649) | *SCN3B,DMD* | 0.04 |
| Head morphogenesis(GO:0060323) | *CSRNP1,ARID5B* | 0.04 |
| Negative regulation of neuron death(GO:1901215) | *PRDX2,NR4A2,NES* | 0.04 |
| hydrolase activity, acting on carbon-nitrogen (but not peptide) bonds(GO:0016810) | *SIRT3,AMPD3,HDAC9* | 0.04 |
| Proteinaceous extracellular matrix(GO:0005578) | *CILP2,ADAMTS8,COL22A1* | 0.04 |
| Cell junction organization(GO:0034330) | *ARHGEF6,RHOD,PVR* | 0.04 |
| Chromosome organization(GO:0051276) | *MEIOB,MYC,TNKS1BP1* | 0.04 |
| Keratinocyte proliferation(GO:0043616) | *SDR16C5,FST* | 0.05 |
| Response to UV(GO:0009411) | *USP28,ERCC5,MYC* | 0.05 |
| Sarcomere organization(GO:0045214) | *FHOD3,ANKRD1* | 0.05 |
| Metanephric nephron development(GO:0072210) | *EGR1,ADIPOQ* | 0.05 |

**Supplementary Table 4.** RIF prediction for regulatory factors in the dataset of DE genes.

| Gene | RIF1 | RIF2 |
| --- | --- | --- |
| *AEBP2* | -4.82 | -1.05 |
| *AFF1* | 0.60 | -1.28 |
| *AFF4* | 0.38 | -2.15 |
| *AHCTF1* | -0.27 | -1.58 |
| *AKAP8* | -2.31 | 0.03 |
| *ANAPC2* | -1.47 | 1.13 |
| *ARID5B* | -0.10 | -2.40 |
| *ASH1L* | -1.11 | -0.45 |
| *ATF2* | -0.67 | -0.44 |
| *ATF3* | -0.05 | -2.23 |
| *ATF5* | -3.39 | -1.30 |
| *ATF6* | 0.22 | -2.06 |
| *ATXN7* | -0.19 | 0.47 |
| *BCLAF1* | -0.61 | -2.38 |
| *BDP1* | 0.01 | -1.26 |
| *BPNT1* | -1.34 | 0.37 |
| *C20orf194* | -4.37 | -1.37 |
| *CARHSP1* | -2.99 | -1.48 |
| *CASZ1* | -2.50 | -1.16 |
| *CBLL1* | 0.54 | -2.07 |
| *CCDC71* | -0.29 | -1.88 |
| *CDC5L* | -1.32 | 0.89 |
| *CEBPG* | -1.58 | -2.52 |
| *CEBPZ* | 0.34 | -1.75 |
| *CHD1* | -0.04 | -2.18 |
| *CHD6* | -2.09 | -0.08 |
| *CLOCK* | -1.21 | -2.33 |
| *COPS2* | -0.19 | -1.69 |
| *CREB1* | -1.44 | -1.29 |
| *CREB5* | -1.15 | -1.80 |
| *CREBL2* | 0.18 | -1.41 |
| *CREM* | 0.38 | -2.22 |
| *CUL1* | 0.14 | -1.74 |
| *CUX1* | -0.19 | -1.63 |
| *DACH1* | -0.04 | -1.25 |
| *DDIT3* | 0.03 | -1.75 |
| *DHX57* | 0.01 | -1.60 |
| *DPF2* | -2.67 | -0.51 |
| *DSP* | -1.87 | -1.61 |
| *DUSP12* | -3.23 | -1.96 |
| *DZIP1* | -0.79 | -1.51 |
| *E2F6* | -1.79 | -1.65 |
| *EBF1* | -1.49 | 0.19 |
| *EGR1* | -0.30 | -2.18 |
| *EGR2* | -0.23 | -2.32 |
| *EGR3* | -1.70 | -2.00 |
| *ELK1* | -1.35 | 0.51 |
| *ELK3* | -1.70 | -1.18 |
| *EOMES* | -3.79 | -1.25 |
| *EP400* | -4.00 | -0.97 |
| *ETS2* | 0.42 | -1.88 |
| *ETV3* | -0.08 | -2.07 |
| *EZH2* | -1.11 | -1.76 |
| *FOS* | 0.00 | -1.87 |
| *FOSL1* | -2.64 | -0.41 |
| *FOSL2* | 0.06 | -2.38 |
| *FOXF1* | -1.64 | -0.10 |
| *FOXJ3* | -1.87 | 0.11 |
| *FOXK1* | -1.92 | 0.04 |
| *FOXK2* | -1.78 | 0.36 |
| *GABPA* | -1.18 | -0.31 |
| *GATAD2A* | -2.28 | -2.42 |
| *GATAD2B* | -4.34 | -2.06 |
| *GMEB1* | 0.01 | -2.09 |
| *GMEB2* | -1.90 | 0.65 |
| *GRHL1* | -1.20 | -1.21 |
| *GTF2F2* | -1.40 | -2.01 |
| *GZF1* | -1.15 | -1.24 |
| *HBP1* | 0.14 | -2.06 |
| *HELZ* | -0.19 | -1.80 |
| *HIF3A* | -1.59 | -0.24 |
| *HIST1H1D* | -2.26 | 0.24 |
| *HMBOX1* | -0.61 | -0.33 |
| *HMG20A* | 0.51 | -0.32 |
| *HMG20B* | -2.46 | -1.57 |
| *HOXD1* | -1.36 | -0.49 |
| *HP1BP3* | -2.36 | -2.14 |
| *HSF2* | -3.09 | -2.01 |
| *IKZF5* | 0.05 | -1.61 |
| *IRF1* | -1.13 | -2.18 |
| *JAZF1* | -1.33 | 0.10 |
| *JUNB* | 0.60 | -1.34 |
| *KAT5* | 0.24 | -1.52 |
| *KCMF1* | -2.27 | -2.42 |
| *KLF11* | -2.30 | -0.92 |
| *KLF12* | -3.66 | 0.10 |
| *KLF15* | -2.90 | -0.05 |
| *KLF2* | -1.41 | 0.83 |
| *KLF5* | 0.24 | -1.81 |
| *KLF6* | -0.09 | -2.26 |
| *KLF9* | -1.40 | -0.30 |
| *LARP4* | -0.01 | -2.04 |
| *LARP7* | -1.85 | -0.86 |
| *LCOR* | -4.07 | -0.45 |
| *LCORL* | -1.31 | 1.03 |
| *LGR4* | -1.63 | -1.02 |
| *MAX* | -1.29 | -0.34 |
| *MGA* | -1.60 | 0.03 |
| *MGMT* | -0.50 | -1.37 |
| *MIER1* | -1.20 | -0.06 |
| *MIER2* | 0.15 | -1.53 |
| *MIER3* | 0.37 | -2.20 |
| *MKRN1* | -2.22 | -2.10 |
| *MLLT3* | -5.35 | -2.31 |
| *MSC* | -0.72 | -1.51 |
| *MYC* | -1.70 | -1.91 |
| *MYF5* | -0.18 | -2.24 |
| *MYF6* | 0.10 | -2.33 |
| *MYNN* | -0.48 | -1.52 |
| *MYOG* | 0.21 | -1.41 |
| *NCOA1* | -0.85 | -1.43 |
| *NCOA2* | -1.25 | -1.15 |
| *NCOA3* | -0.23 | -1.58 |
| *NCOR1* | -2.95 | 0.65 |
| *NFAT5* | -2.21 | -1.27 |
| *NFE2* | -2.78 | -1.03 |
| *NFE2L3* | -2.86 | -0.66 |
| *NFIA* | -2.98 | -1.08 |
| *NFIL3* | 0.16 | -2.19 |
| *NFKB1* | 0.23 | -2.32 |
| *NFRKB* | -2.90 | -2.17 |
| *NFXL1* | 1.03 | -1.37 |
| *NFYA* | -0.30 | -0.30 |
| *NKRF* | 0.09 | -1.90 |
| *NKX6-2* | 0.11 | -2.18 |
| *NOC3L* | -0.30 | -1.89 |
| *NOTO* | -1.81 | -1.45 |
| *NR0B2* | 0.78 | -1.27 |
| *NR1D2* | -1.06 | -2.26 |
| *NR2C1* | -0.78 | -1.70 |
| *NRF1* | -2.29 | -0.36 |
| *NUFIP1* | 0.18 | -2.16 |
| *OSR1* | -2.05 | 0.17 |
| *PATZ1* | 0.93 | -1.37 |
| *PBRM1* | -0.73 | -1.42 |
| *PBX3* | -2.13 | 0.16 |
| *PCGF6* | -0.47 | -2.17 |
| *PCSK6* | -2.01 | -0.16 |
| *PDS5B* | -0.32 | -2.22 |
| *PHF20* | 0.03 | -1.83 |
| *PKNOX1* | -1.00 | 1.26 |
| *PLAGL2* | -0.28 | -2.03 |
| *PLXNB1* | -1.54 | -0.16 |
| *PMS1* | -1.32 | -1.97 |
| *POGK* | -0.29 | -1.40 |
| *POLE3* | -2.13 | -1.14 |
| *POU2F1* | -3.18 | -1.85 |
| *POU3F1* | -1.46 | -0.81 |
| *PPARD* | 0.27 | -1.31 |
| *PRDM1* | -1.42 | -1.85 |
| *PRDM12* | -1.24 | -1.13 |
| *PRDM8* | -1.71 | -1.63 |
| *PSMD12* | -0.06 | -2.25 |
| *RBM10* | -1.70 | -2.25 |
| *RBM20* | -0.21 | -0.71 |
| *RBM22* | 0.35 | -2.11 |
| *RBM26* | -1.02 | -2.29 |
| *RBM5* | -2.36 | 0.33 |
| *RBPJ* | -1.23 | -1.40 |
| *RC3H1* | 0.17 | -1.59 |
| *RC3H2* | -0.13 | -1.38 |
| *RCOR3* | -2.68 | -1.95 |
| *RELA* | -2.86 | -1.20 |
| *REST* | -2.86 | -1.01 |
| *RFX2* | -1.13 | -1.87 |
| *RFX3* | -1.87 | -1.06 |
| *RFX5* | -1.89 | -1.92 |
| *RFX7* | -1.47 | -0.26 |
| *RGS7* | -1.26 | 0.51 |
| *RLF* | -0.44 | 0.73 |
| *RNF114* | -2.63 | -1.61 |
| *RNF138* | -0.87 | -1.79 |
| *RUNX1* | -0.17 | -2.27 |
| *SATB2* | -2.50 | -0.48 |
| *SF3A2* | -0.50 | -2.12 |
| *SF3A3* | -0.10 | -1.76 |
| *SLC39A10* | 0.67 | -1.69 |
| *SMAD1* | -0.45 | -2.20 |
| *SMAD3* | -0.17 | -2.31 |
| *SMAD4* | -2.20 | -2.35 |
| *SMAD5* | -1.06 | -2.21 |
| *SMARCA1* | -0.63 | -1.99 |
| *SMARCC2* | -1.18 | -1.56 |
| *SMARCE1* | -0.01 | -1.39 |
| *SNAPC4* | -1.64 | 0.16 |
| *SOX6* | -2.29 | -0.10 |
| *SP1* | 0.57 | -1.61 |
| *SP2* | 0.57 | -1.40 |
| *SSB* | 0.14 | -1.29 |
| *SSH2* | -2.28 | -0.38 |
| *STAT2* | -0.65 | -2.29 |
| *STAT3* | 0.59 | -1.34 |
| *STAT5A* | 0.30 | -1.42 |
| *SUZ12* | -1.06 | 0.10 |
| *TCF12* | -2.88 | -1.88 |
| *TCF15* | 0.07 | -1.31 |
| *TEAD3* | 0.19 | -1.69 |
| *TEAD4* | -1.03 | -0.88 |
| *TEF* | -2.69 | -1.11 |
| *TGIF1* | -0.58 | -2.42 |
| *TGIF2* | -3.05 | -0.34 |
| *THAP1* | -0.37 | -1.73 |
| *THAP2* | -0.15 | -1.66 |
| *THAP5* | -0.03 | -2.24 |
| *THRB* | -0.32 | -0.74 |
| *TIPARP* | -0.51 | -1.79 |
| *TOX4* | 0.12 | -1.40 |
| *TP63* | -2.63 | -2.24 |
| *TRAFD1* | -0.23 | -2.33 |
| *TRMT1* | -4.40 | -0.67 |
| *TSC22D2* | -0.79 | 0.69 |
| *TTF1* | -1.82 | 0.12 |
| *UBE2K* | -0.05 | -2.41 |
| *UBP1* | -0.83 | -1.35 |
| *USP39* | -1.87 | -0.42 |
| *VDR* | -2.24 | -2.12 |
| *XPA* | -2.42 | -2.18 |
| *YOD1* | -0.67 | -1.86 |
| *ZBED4* | -2.99 | 0.54 |
| *ZBTB10* | 0.14 | -2.09 |
| *ZBTB16* | -2.77 | -1.77 |
| *ZBTB17* | -1.33 | -1.63 |
| *ZBTB20* | -1.75 | 0.83 |
| *ZBTB24* | -0.79 | -1.98 |
| *ZBTB3* | -3.02 | -2.00 |
| *ZBTB34* | -0.06 | -2.40 |
| *ZBTB38* | -2.02 | -0.05 |
| *ZBTB39* | 0.16 | -2.24 |
| *ZBTB41* | 0.07 | -1.96 |
| *ZBTB44* | -3.05 | -0.21 |
| *ZBTB45* | -2.15 | 0.11 |
| *ZBTB47* | -1.81 | -2.09 |
| *ZC3H10* | -0.33 | -2.27 |
| *ZC3H13* | -1.69 | -0.59 |
| *ZC3H4* | -1.57 | 0.35 |
| *ZEB1* | -2.22 | 0.17 |
| *ZEB2* | -1.78 | -1.23 |
| *ZFHX4* | -1.38 | 0.78 |
| *ZFP1* | -0.03 | -2.36 |
| *ZFP28* | -1.98 | -0.97 |
| *ZFP36L2* | -1.42 | -2.28 |
| *ZFP62* | -1.98 | 0.49 |
| *ZFP90* | -2.27 | 0.15 |
| *ZFPM2* | -3.67 | -0.56 |
| *ZFR* | -0.74 | -2.34 |
| *ZFYVE26* | -3.50 | -1.72 |
| *ZIC4* | -3.52 | -1.79 |
| *ZNF143* | -0.13 | -2.19 |
| *ZNF182* | -3.48 | -1.13 |
| *ZNF202* | -0.95 | -2.37 |
| *ZNF236* | -0.18 | -2.11 |
| *ZNF251* | -2.87 | -2.02 |
| *ZNF268* | -2.37 | 0.40 |
| *ZNF275* | 0.79 | -1.39 |
| *ZNF280B* | -0.18 | -1.99 |
| *ZNF280C* | -1.59 | -1.71 |
| *ZNF280D* | -0.55 | -1.35 |
| *ZNF281* | -0.21 | -2.19 |
| *ZNF282* | -0.66 | -1.48 |
| *ZNF283* | -1.44 | 0.62 |
| *ZNF300* | -1.36 | 0.20 |
| *ZNF316* | -1.76 | -1.50 |
| *ZNF318* | -1.06 | -1.44 |
| *ZNF326* | -1.45 | -1.54 |
| *ZNF335* | -1.42 | 0.59 |
| *ZNF354A* | -0.36 | -1.91 |
| *ZNF366* | -1.44 | 0.80 |
| *ZNF367* | -4.85 | -1.57 |
| *ZNF407* | 0.07 | -1.27 |
| *ZNF410* | -3.04 | 0.17 |
| *ZNF43* | -1.41 | 0.49 |
| *ZNF451* | -2.23 | -1.81 |
| *ZNF462* | -4.65 | -1.61 |
| *ZNF48* | -0.55 | -2.20 |
| *ZNF501* | -0.08 | -1.53 |
| *ZNF507* | 0.56 | -2.32 |
| *ZNF532* | -2.14 | 0.41 |
| *ZNF546* | -0.34 | -1.30 |
| *ZNF565* | -3.18 | -0.39 |
| *ZNF568* | -0.48 | -1.42 |
| *ZNF572* | -0.96 | -2.04 |
| *ZNF580* | -0.98 | -1.69 |
| *ZNF619* | -2.44 | -1.07 |
| *ZNF624* | -1.37 | 0.40 |
| *ZNF638* | -1.29 | 1.04 |
| *ZNF641* | -2.05 | -1.84 |
| *ZNF644* | -0.51 | -1.88 |
| *ZNF658* | -2.33 | -0.94 |
| *ZNF662* | -1.43 | -0.82 |
| *ZNF671* | -1.82 | -0.67 |
| *ZNF672* | -0.46 | 0.26 |
| *ZNF697* | -0.47 | -2.41 |
| *ZNF71* | -2.61 | -0.62 |
| *ZNF746* | -2.65 | 0.34 |
| *ZNF75D* | -1.38 | -0.92 |
| *ZNF789* | -1.31 | -1.38 |
| *ZNF793* | -1.43 | 0.00 |
| *ZNF800* | -0.73 | -2.30 |
| *ZSCAN12* | -2.84 | -1.76 |
| *ZUFSP* | 0.32 | -1.80 |
| *ZZZ3* | -0.33 | -2.26 |

^¥^Bootstrap 99% confidence intervals for RIF1 z-scores: −1.193/2.352

^†^Bootstrap 99% confidence intervals for RIF2 z-scores: −1.223/1.648

**Supplementary Table 5.** Potential transcription factors (TRF) with extreme RIF z-scores and identified by IPA software and their corresponding target DE genes.

| TRF | Target genes in the differentially expressed dataset |
| --- | --- |
| ATF3 | PPP1R15A (NW2) |
| ATF6 | FOS (NW3),PRDX2 (NW3) |
| CLOCK | ELOVL6 (NW3), FASN (NW3),GADD45A (NW2), GADD45B (NW2), JUNB (NW3) |
| CREB1 | ESRRG (NW2), FASN (NW3), FOS (NW3), FOSB,GADD45A (NW2),GADD45B (NW2),GADD45G (NW2),IGSF9B,JUNB (NW1),KCNC4,MYC,NR4A2,PEG10,PPP1R15A (NW2), RPRM (NW1),SCD (NW3),SCN3B,TRAFD1 (NW2) |
| CREM | ACTC1 (NW1),ATF3,CSRNP1,DUSP1,EGR1 ,EGR2 ,FOS (NW3),GADD45B (NW2),JUNB (NW3),NR4A2,PPP1R15A (NW2) |
| DACH1 | EGR1, FOS (NW3) |
| DDIT3 | ANKRD1 (NW2),ATF3,PPP1R15A (NW2) |
| EBF1 | ADIPOQ, FASN (NW3),SCD (NW3) |
| EGR1 | ATF3,EGR1 ,EGR2 ,GADD45A (NW2),GADD45B (NW2),HBEGF,JUNB (NW3),MYC,PEG10,SPP1 |
| EGR2 | EGR1 ,EGR2 ,FOS (NW3),SCD (NW3) |
| ELK1 | EGR1 ,EGR2 ,FOS (NW3),JUNB (NW3),SPP1 |
| ELK3 | EGR1 ,FOS (NW3) |
| ETS2 | FOS (NW3),JUNB (NW3),MYC,SPP1 |
| ETV3 | MYC,SPP1 |
| FOS | ATF3,CTGF ,EGR1 ,EGR2 ,ELOVL6 (NW3),ERCC5 (NW2),FASN (NW3),FOS (NW3),FOS (NW3)B,JUNB (NW3),MYC,SCD (NW3),SEMA4D (NW3),SLPI,SEMA4D (NW3),SPP1,TNMD |
| FOSL1 | EGR1 ,EGR2 ,FOS (NW3),FOSB (NW3),JUNB (NW3) |
| JUNB | ATF3,DUSP1,FASN (NW3),JUNB (NW3),TNMD (NW1),PLIN1 (NW3),SCD (NW3) |
| KLF15 | CTGF ,DGAT2 (NW3),ESRRG (NW2),FASN (NW3),SCD (NW3) |
| KLF2 | CTGF ,GADD45A (NW2),JUNB (NW3),MYC |
| KLF5 | DUSP1,MYC |
| MAX | ACTC1 (NW1),GADD45A (NW2),GADD45B (NW2),MYC |
| MSC | AMPD3,GPAT3 (NW3),SPP1 |
| MYC | CIDEC (NW3),DUSP1,EGR1 ,EGR2 ,FASN (NW3),FOS (NW3),GADD45A (NW2),GADD45B (NW2),GADD45G (NW2),IER5,MYC,PEG10,PFKFB3 ,PRDX2 (NW3),SPP1 |
| MYOG | FOS (NW3),MYF6 (NW2) |
| NCOA1 | ADIPOQ ,MYC,THRSP (NW3) |
| NCOA2 | ADIPOQ ,EGR1 ,FASN (NW3),FST (NW2),PLIN1 (NW3) |
| NCOR1 | ADIPOQ ,DUSP1,FASN (NW3),PLIN1 (NW3),THRSP (NW3) |
| NFIL3 | FASN (NW3),GADD45A (NW2),GADD45B (NW2),SCD (NW3) |
| NFKB1 | ANKRD1 (NW2),CTGF ,CYR61 ,DUSP1,EGR1 ,FOS (NW3),FOSB (NW3),MYC,NR4A2 |
| POU2F1 | GADD45A (NW2),PRDX2 (NW3),SPP1 |
| PRDM1 | CA9 (NW2),EGR2 ,FOS (NW3),GPAT3 (NW3),MYC |
| RELA | ADIPOQ ,ANKRD1 (NW2),CTGF ,DUSP1,EGR1 ,EWSR1,FOS (NW3),FOS (NW3)B,JUNB (NW3),MYC,NR4A2 |
| RFX3 | MAP1A,MYC |
| SMAD1 | CTGF ,GADD45B (NW2),MYC,SPP1 |
| SMAD3 | ANKRD1 (NW2),CTGF ,EGR1 ,FOS (NW3),FST (NW2),GADD45B (NW2),HBEGF,JUNB (NW3),MYC,SPP1 |
| SMAD4 | CTGF ,ERCC5 (NW2),FOS (NW3),FST (NW2),GADD45A (NW2),GADD45B (NW2),MYC,PTK2B,SCD (NW3) |
| SMARCA1 | GADD45A (NW2),MYC |
| SMARCE1 | FOS (NW3),MYC |
| SP1 | ATF3,CTGF ,DMD (NW1),EGR1 ,FASN (NW3),FOS (NW3),HBEGF,MYC,NES ,SLC4A7 ,SEMA4D (NW3),SPP1 |
| STAT3 | ADIPOQ ,AHSP (NW1),CTGF ,EGR1 ,EGR2 ,EGR3 (NW3),FASN (NW3),FOS (NW3),FST (NW2),GADD45A (NW2),GADD45G (NW2),JUNB (NW3),MYC,NES ,NR4A2,PEG10 |
| STAT5A | ADIPOQ ,CISH,FASN (NW3),FOS (NW3),FOS (NW3)B,GADD45A (NW2),GADD45G (NW2),MYC,NR4A2,TNNI1 |
| TP63 | CYR61 ,ERCC5 (NW2),FASN (NW3),FOS (NW3),FST (NW2),GADD45A (NW2),HBEGF,JUNB (NW3),MYC |
| VDR | ADIPOQ ,GADD45A (NW2),SPP1 |
| ZBTB20 | CISH,FASN (NW3) |

IPA network of several target genes. Network 1 (NW1); network 2 (NW2) and network 3 (NW3)
