# Supplementary figures and images for: Identification of Candidate Genes and Regulatory Factors Underlying Intramuscular Fat Content Through Longissimus Dorsi Transcriptome Analyses in Heavy Iberian Pigs
Source: Front Genet. 2018 Dec 4;9:608. doi: 10.3389/fgene.2018.00608 (PMC6288315; doi:10.3389/fgene.2018.00608)

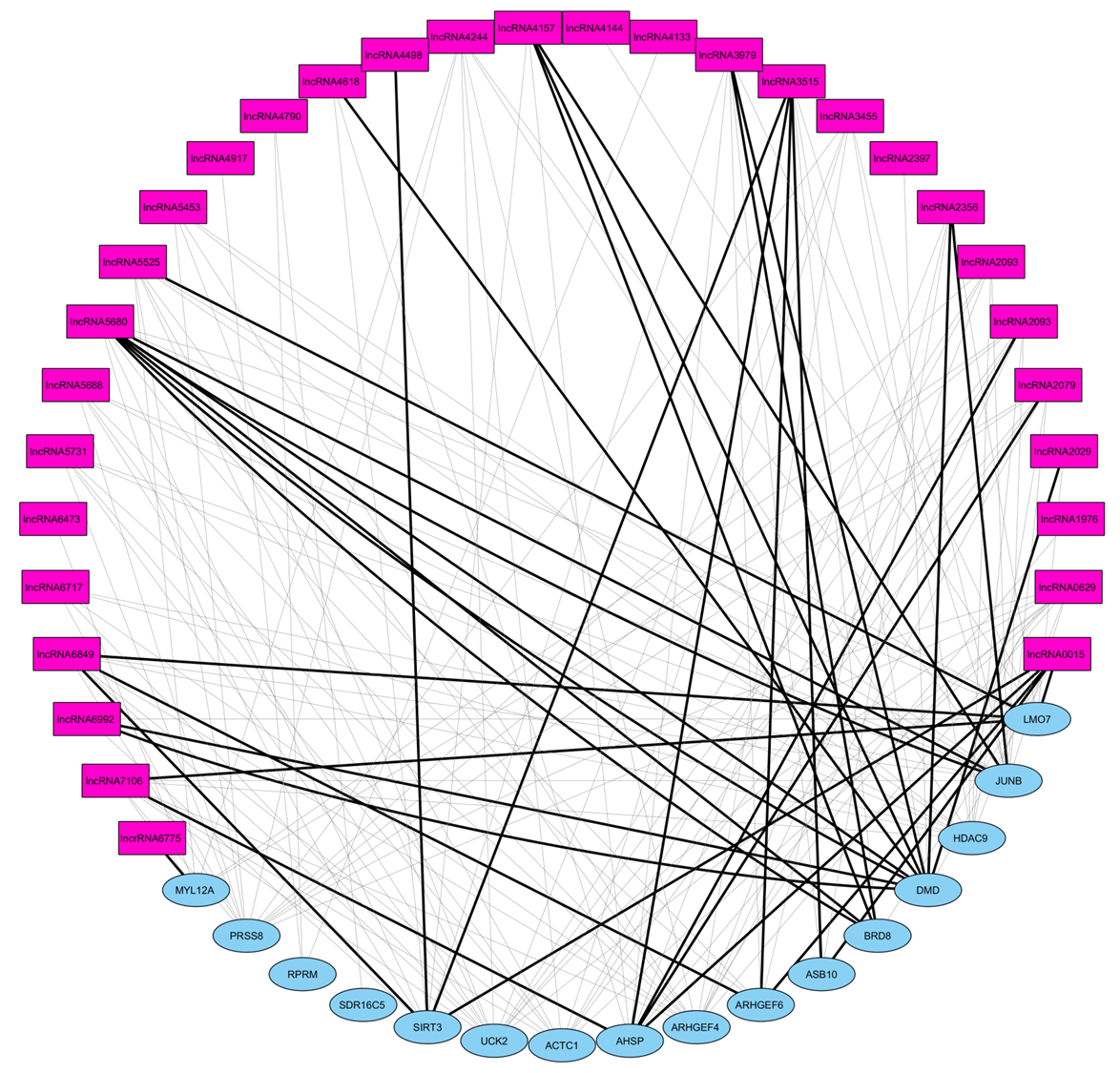

Supplement: Supplementary file 2 [file Image_1.TIF]

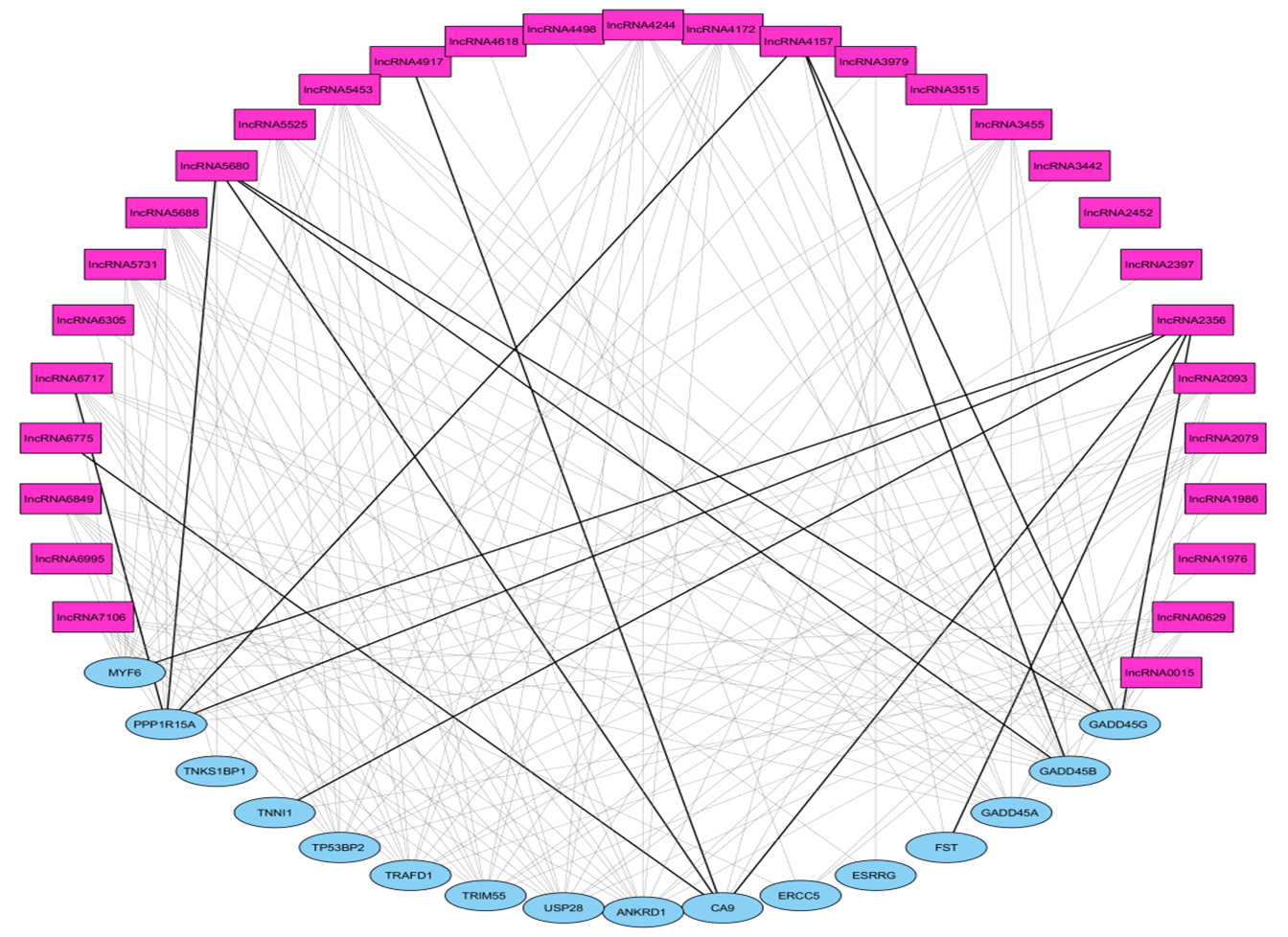

Supplement: Supplementary file 3 [file Image_2.TIF]

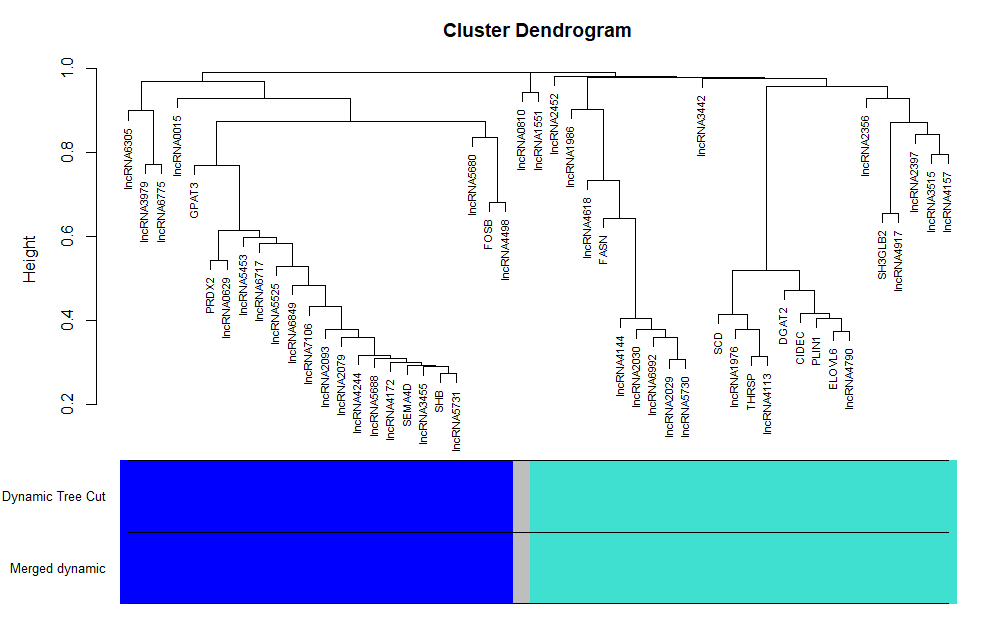

Supplement: Supplementary file 4 [file Image_3.TIFF]

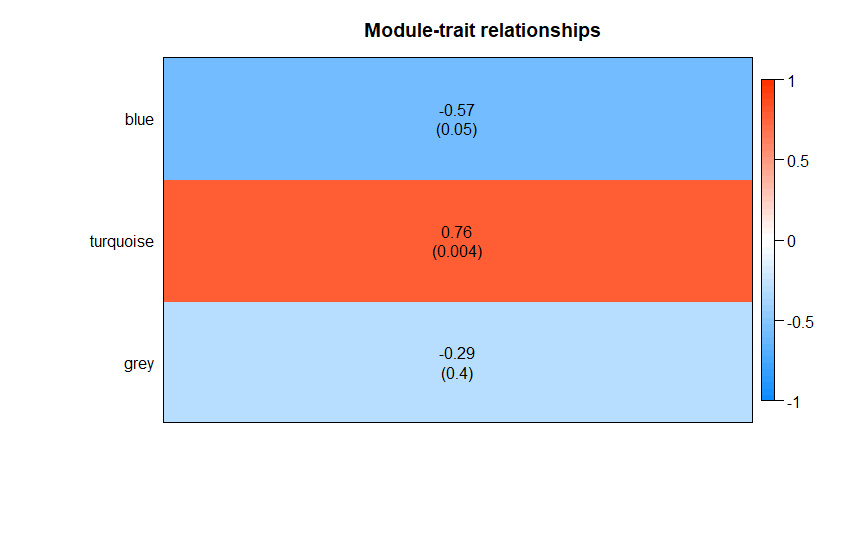

Supplement: Supplementary file 5 [file Image_4.TIFF]
